# A user’s guide for understanding reptile and amphibian hydroregulation and climate change impacts

**DOI:** 10.1093/conphys/coaf038

**Published:** 2025-06-16

**Authors:** Nicholas C Wu, Rodolfo O Anderson, Amaël Borzée, Shannon Buttimer, Mathias Dezetter, Shahar Dubiner, Quan-Heng Li, Carlos A Navas, Daniel Sánchez-Ochoa, Jennifer A Sheridan, Swapnil A Shewale, Bao-Jun Sun, Sunil J Suryawanshi, Jia-Huan Wang, Rafael P Bovo

**Affiliations:** Centre for Terrestrial Ecosystem Science and Sustainability, Harry Butler Institute, Murdoch University, Murdoch, 90 South Street, WA 6150, Australia; School of Environmental and Conservation Science, Murdoch University, Murdoch, 90 South Street, WA 6150, Australia; School of Biological Sciences, Monash University, 25 Rainforest Walk, Melbourne, VIC 3800, Australia; Laboratory of Animal Behaviour and Conservation, College of Life Sciences, Nanjing Forestry University, No. 159, Xinzhuang, Longpan Road, Nanjing 210037, China; Department of Biology, The Pennsylvania State University, 168 Curtin Rd, University Park, PA 16802, USA; Nature Conserv'Action, 28 Avenue Georges Clemenceau, Saint-Jean-de-Védas, Montpellier 34430, France; School of Zoology, Faculty of Life Sciences, Tel Aviv University, Klausner Street, Tel Aviv 6997801, Israel; Key Laboratory of Animal Ecology and Conservation Biology, Institute of Zoology, Chinese Academy of Sciences, 1 Beichen West Road, Chaoyang District, Beijing 100101, China; Departmento de Fisiologia, Instituto de Biociências, Universidade de São Paulo, Rua do Matão - Travessa 14 - N. 101 Cidade Universitária - Cep. 05508-090, SP, Brazil; Facultad de Estudios Superiores Iztacala, Universidad Nacional Autónoma de México, Tlalnepantla de Baz 54090, México; Carnegie Museum of Natural History, 4400 Forbes Ave, Pittsburgh, PA 15213, USA; Department of Zoology, Bharatiya Vidya Bhavan's Hazarimal Somani College, Chowpatty, Mumbai 400007, India; Key Laboratory of Animal Ecology and Conservation Biology, Institute of Zoology, Chinese Academy of Sciences, 1 Beichen West Road, Chaoyang District, Beijing 100101, China; Yashavantrao Chavan Institute of Science, Karmaveer Bhaurao Patil University, Powai Naka, Satara, Maharashtra 415001, India; Key Laboratory of Animal Ecology and Conservation Biology, Institute of Zoology, Chinese Academy of Sciences, 1 Beichen West Road, Chaoyang District, Beijing 100101, China; Department of Evolution, Ecology, and Organismal Biology, University of California Riverside, Eucalyptus Dr #2710, Riverside, CA 92521, USA; Departamento de Biologia, Faculdade de Filosofia, Ciências e Letras de Ribeirão Preto, Universidade de São Paulo, Av. Bandeirantes 3900, Ribeirão Preto, SP 14040-901, Brazil

**Keywords:** Dehydration, drought, ectotherm, exposure, sensitivity, vulnerability, water balance

## Abstract

Human impacts on ecosystems have intensified variation in water variability for terrestrial life, thus challenging the maintenance of water balance, or hydroregulation. The accelerated development and accessibility of technologies and computational models over the past decade have enabled researchers to predict changes in animal hydroregulation and environmental water with greater spatial and temporal precision. Focusing on reptiles and amphibians, we discuss current methods, limitations and advances for quantifying ecologically relevant metrics of environmental water stressors and organismal responses to both acute and long-term water stress that are applicable for conservation and management. We also highlight approaches that integrate environmental water data with an organism’s water balance and physiological, behavioural and life history traits to predict the limits of species’ responses and assess their vulnerability to climate change. Finally, we outline promising future directions and opportunities in hydroregulation studies with a conservation focus, including broader inferences about acclimation responses, linking gene expression to functional changes, and exploring inter- and transgenerational plasticity and adaptive evolution. Advances in these fields will facilitate more accurate assessments of species’ capacities and the limits of hydroregulation in response to a more variable and unpredictable future climate.

##  Introduction

**Figure 1:**
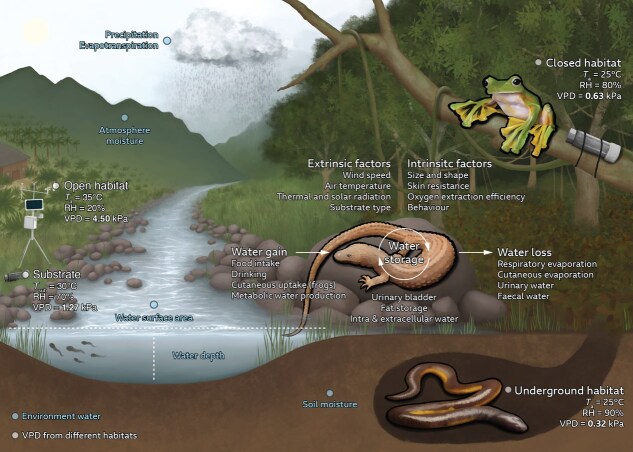
**Overview of the landscape hydrology and animal hydroregulation**. Blue text indicates environmental water that can influence hydroregulation such as precipitation and evapotranspiration, atmospheric and soil moisture content, water bodies (outlined in Table 1) and their interaction with external factors such as wind speed, temperature, thermal radiation and substrate composition. The landscape includes habitats with different water stressors represented by VPD (in kilopascals), which is calculated from measured air temperature (in degrees Celsius) and atmospheric moisture content (e.g. RH in %). Hydroregulation includes water gain/loss, water storage and their interaction with extrinsic and intrinsic factors. Representative landscape and animals are based on Borneo’s ecosystem. The representative terrestrial lizard is the earless monitor lizard (*Lanthanotus borneensis*), the representative arboreal frog is the Wallace’s flying frog (*Rhacophorus nigropalmatus*) and the representative subterranean caecilian is the Metang caecilian (*Ichthyophis biangularis*). Illustration by S. Buttimer.

The colonization of land presented substantial physiological and morphological challenges associated with water balance for early terrestrial animals (Gray, 1928; Dial *et al.*, 2015), yet it allowed opportunities for novel evolutionary strategies that enabled an explosion of animal diversity on land (Minter *et al.*, 2017). Nevertheless, environmental dryness continues to pose a major challenge for land animals, with biodiversity being highest in humid tropical rainforests and lowest in desert environments (Owen, 1989; Biber *et al.*, 2023; Coelho *et al.*, 2023). Global trends towards aridification can therefore compromise biodiversity, a major conservation concern given accelerated changes in climate and land use, leading to unpredictable changes in water variability and availability (Moustakis *et al.*, 2021; Moss *et al.*, 2024; Zhang *et al.*, 2024). Understanding (1) how environmental water changes over time and space, (2) how animals respond to water variability and (3) how they differ in resilience and response capacity is necessary to assess vulnerability, and it is a first step in managing extinction risk amid the current global biodiversity crisis. Progress towards these answers has been notable, due to enhanced computational power, novel statistical models and more temporal and spatially detailed climate data (Brun *et al.*, 2022; Klinges *et al.*, 2024). Additionally, our understanding of hydroregulation strategies across biological levels and species has become more comprehensive (Navas and Carvalho, 2010; Lillywhite, 2016; Rozen-Rechels *et al.*, 2019; Riddell *et al.*, 2021). A key challenge for the management and conservation of species at risk due to climate change is to predictively link ecologically relevant water stressors (exposure risk) with the capacity of animals to maintain water balance (species sensitivity) (Fig. 1).

This paper reviews current knowledge on terrestrial water availability for assessing environmental exposure risk, species’ short- and long-term responses to water deficits for evaluating sensitivity, and models predicting vulnerability to environmental drying. We examine key topics and conclude proposing future directions for refining predictions of species’ vulnerability in a drying world. Our focus on amphibians and reptiles highlights their role in the aquatic–terrestrial transition and the contrasting hydroregulation strategies that enable them to thrive and reproduce despite dehydration challenges. Also, there are already great reviews published for insects (Chown *et al.*, 2011; Sinclair *et al.*, 2024) and for endotherms, which are tightly linked to their thermoregulation (Mitchell *et al.*, 2018; McKechnie and Wolf, 2019).

## Environmental Exposure Risk

### Water availability on land

Water availability can be quantified and interpreted in many ways, and it is heavily influenced by the stochastic nature of the water cycle (Chahine, 1992; Oki and Kanae, 2006) and by the local environment (Kinlaw, 1999; Geiger *et al.*, 2003). Broadly, water enters a terrestrial environment through precipitation, and exits through evapotranspiration and run-off (Oki and Kanae, 2006). The ratio between precipitation and evapotranspiration can be used to calculate the aridity index (Zomer *et al.*, 2022), broadly define climate classifications (Beck *et al.*, 2018) and quantify annual site water balance. In climates with strong seasonal rainfall, precipitation can predict the phenology of breeding events (Gould *et al.*, 2022; Thompson *et al.*, 2022) and the seasonal primary productivity of ecosystems (Lieth, 1973). Once in the environment, water can be stored in the ground, in the air or accumulated in water bodies (Table 1). Water content in the air is typically measured as the pressure of gaseous water, or water vapour pressure (Gates, 1980), which serves as a basis for calculating relative humidity (RH), a common meteorological metric. However, RH is often less relevant for assessing physiological responses in organisms compared to water vapour pressure (Anderson, 1936; Kurta, 2014; Wu *et al.*, 2024b). Finally, water vapour pressure and temperature are inherently linked (Campbell and Norman, 2000), and incorporating temperature allows for the calculation of vapour pressure deficit (VPD), a key driver of physiological processes in plants and animals (Adolph, 1932; Novick *et al.*, 2024; Wu *et al.*, 2024a). For example, in two environments with the same humidity, that with higher air temperatures will increase VPD (Fig. 1).

**Table 1:** Environmental water variables and example indices of environmental dryness with definitions and calculations of the variables, their interpretation for reptiles and amphibians, the temporal resolution scale available and non-exhaustive examples of online global datasets to extract environmental water variables and indices

Name	Definition and calculation	Interpretation	Resolution	Example global database
**Environmental water**				
Precipitation (*P*)	Amount of rainfall per unit area (mm or kg m^2^).	Less rainfall = less water available for animals to rehydrate.	Hourly to yearly	*CHIRPS*: Global daily rainfall from 1981 to near-present (Funk *et al.*, 2015). *WorldClim 2*: Average yearly precipitation and seasonality from 1970 to 2000 (Fick and Hijmans, 2017). *CHELSA*: Precipitation, and potential evapotranspiration (1981–2010) with future scenarios at three future time periods (2011–40, 2041–70 and 2071–2100) under three shared socioeconomic pathways (SSP126, SSP370, SSP585) and across five Earth system models (Brun *et al.*, 2022). *CHELSA-EarthEnv*: Global daily rainfall from 2003 to near-present (Karger *et al.*, 2021). *TerraClim*: Average (1958–2019) global rainfall with future scenarios of +2°C and +4°C (Abatzoglou *et al.*, 2018).
Atmospheric moisture content (RH or *e*)	Amount of moisture (water vapour) the air holds. Typically expressed as RH (%), absolute humidity (g cm^−3^), or actual vapour pressure (*e*_a_; kPa)	Less moisture in the air = higher evaporation of water through evaporative surfaces.	Seconds to yearly	*CHELSA*: Near-surface RH (1981–2010) with future scenarios at three future time periods (2011–40, 2041–70 and 2071–2100) under three shared socioeconomic pathways (SSP126, SSP370, SSP585) and across five Earth system models (Brun *et al.*, 2022). *TerraClim*: Average (1958–2019) global vapour pressure with future scenarios of +2°C and +4°C (Abatzoglou *et al.*, 2018). *MODIS*: 5-min interval global water vapour data (https://ladsweb.modaps.eosdis.nasa.gov/missions-and-measurements/science-domain/water-vapor/#modis). *MODIS*: 8-day to annual interval global evapotranspiration data (https://ladsweb.modaps.eosdis.nasa.gov/missions-and-measurements/science-domain/evapotranspiration/).
Soil moisture content (Φ)	Amount of water the soil holds. Expressed as volume (m^3^), weight (kg) or water potential (kPa or J kg^−1^).	Relevant for animals that use burrows to acquire (from the soil/substrate), conserve (no water exchange) and reduce water loss.	Seconds to yearly	*NicheMapR*: Above- and below-ground microclimate from various sources (Kearney and Porter, 2017). *Microclimc*: Above- and below-ground microclimate (Maclean and Klinges, 2021).

(Continued)

**Table 1:** Continued

Name	Definition and calculation	Interpretation	Resolution	Example global database
Water surface area	Land area covered by freshwater (%) e.g. lakes, rivers.	Amount of large-bodied freshwater sources for animals to rehydrate and/or breed.	Average over set years.	*HYDROSHEDS*: Global hydrographic products such as catchment boundaries, river networks and lakes at multiple resolutions and scales (www.hydrosheds.org).
**Environmental dryness indices**				
Aridity index (AI) or climate moisture index (CMI)	AI = *P*/*PET* CMI (mm or kg m^2^ month^−1^) = *P* − *PET* The difference (CMI) or ratio (AI) between the average annual precipitation (*P*) and potential evapotranspiration (*PET*).	Indicator of the degree of dryness of the climate.	Depending on *P* and *PET* resolution, but typically monthly to yearly average.	*CHELSA*: CMI (1981–2010) with future scenarios at three future time periods (2011–40, 2041–70 and 2071–2100) under three shared socioeconomic pathways (SSP126, SSP370, SSP585) and across five Earth system models (Brun *et al.*, 2022). *Global-AI_PET_v3*: Global hydro-climatic data averaged (1970–2000) monthly and yearly (Zomer *et al.*, 2022).
Drought index	Standardized index representing meteorological drought based on different formulas. Common indices include: PDSI SPI NDVI	Indicator of change in environmental dryness relative to ‘normal’ conditions of the location. The intensity, frequency and duration of drought events can be calculated from these indices.	Monthly to decades.	*TerraClim*: Average (1958–2019) global PDSI with future scenarios of +2 °C and +4 °C (Abatzoglou *et al.*, 2018). *Dai_et_al_2004*: Global PDSI under three shared socioeconomic pathways: 1870–2002, SSP245 and *SSP585* (Dai *et al.*, 2004). *MODIS*: 16-day and monthly interval global NDVI (https://modis.gsfc.nasa.gov/data/dataprod/mod13.php).
VPD	VPD (kPa) = *e*_s_ – *e*_a_ The difference between the amount of moisture in the air (*e*_a_) and how much moisture the air can hold when it is saturated at known temperature (*e*_s_).	Determines desiccation risk and relates to the primary productivity of ecosystems (plant growth, food availability).	Depending on *e*_s_ and *e*_a_ resolution, but typically monthly to yearly average.	*CHELSA*: VPD (1981–2010) with future scenarios at three future time periods (2011–40, 2041–70 and 2071–2100) under three shared socioeconomic pathways (SSP126, SSP370, SSP585) and across five Earth system models (Brun *et al.*, 2022). *TerraClim*: Average (1958–2019) global VPD with future scenarios of +2°C and +4°C (Abatzoglou *et al.*, 2018).

Water in the soil matters most for species that rely on underground microrefugia (Wu *et al.*, 2015; Giacometti and Tattersall, 2023), or for many amphibians, which obtain water directly from the substrate (Hillman *et al.*, 2009; Comanns *et al.*, 2017; Lemenager *et al.*, 2022). Water fluxes depend on soil properties (Campbell and Norman, 2000), with extreme examples in sand and clay. Wet sands have an open texture and dry quickly, whereas wet clays exhibit high soil moisture tension and dry slowly. In comparison, wet peats dry rapidly and are difficult to rehydrate. However, a valid generalization is that below-ground climates maintain higher humidity than surface environments, reducing dehydration risk to animals (Fig. 1), e.g. in species that shelter underground (Carvalho *et al.*, 2010), and especially desert dwellers (Woodbury, 1954; Bulova, 2002).

### Quantifying water variability and drought indices

**Figure 2:**
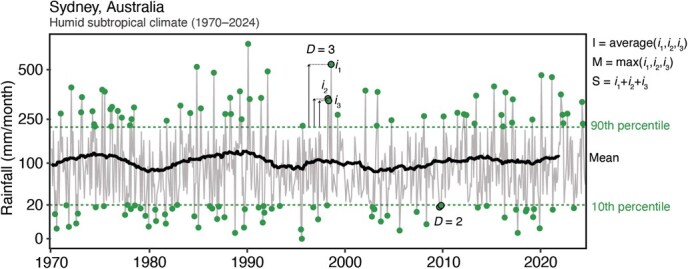
**Example variation in monthly precipitation in Sydney, Australia, from 1970–2024**. Monthly precipitation data represented by thin grey lines from the Australian Government Bureau of Meteorology, with the 5-year rolling mean in thick black lines. The 10th and 90th month-specific percentiles represent dry and wet thresholds, respectively. Example durations (*D*) for extremely dry or wet months (green points with black outline) are shown, which is calculated as the number of consecutive months above the wet and below the dry thresholds. Example calculations of intensity (I), magnitude (M) and severity (S) are also shown for a 3-month wet event (*D* = 3) with a departure of *i*_1_, *i*_2_ and *i*_3_ from the threshold. Frequency can be calculated as the number of times the monthly precipitation is above the wet and below the dry thresholds.

Quantifying spatiotemporal shifts in hydric patterns can be approached in various ways to determine whether an environment is drier than usual. Precipitation and moisture levels, whether in the air or soil, can be measured long-term via weather stations, or short-term using miniature environmental data loggers, with Bramer *et al.* (2018) and De Frenne *et al.* (2025) providing examples of commercially available loggers and field deployment considerations. When long-term datasets are available, various hydrological extreme metrics can be calculated (Pisor *et al.*, 2023). For instance, using a monthly rainfall example from Sydney, Australia (Fig. 2), one can calculate the duration (D) of high rainfall (>90th percentile; high likelihood of flooding) and low rainfall events (<10th percentile; high likelihood of drought), frequency of extreme rainfall events and measures of intensity (I)—the average of extreme rainfall events—magnitude (M)—the maximum rainfall event—and severity (S)—the cumulative total of extreme rainfall events. Changes in permanent water sources such as lakes, ponds, streams and rivers can be quantified via drones (Spence and Mengistu, 2016; Woodget *et al.*, 2017), satellites (Nath and Deb, 2010; Zhou *et al.*, 2021) or directly using standard environmental monitoring tools, enabling spatiotemporal quantification of water body dynamics, particularly relevant for species reliant on temporary or permanent aquatic habitats (Table 1).

Relevant for policy makers, quantifying environmental drying risk requires simplification of complex metrics and variables. One option is relying on meteorological drought indices reflecting the interplay between the climate variables mentioned in the previous section (Table 1). However, theses indices have no absolute value, meaning that changing indices relate to species-specific effects that may change among individuals, populations and communities. The simplest drought index is the Standardized Precipitation Index (SPI), which relies only on precipitation data (McKee *et al.*, 1993). Complex counterparts include the Palmer Drought Severity Index (PDSI), which incorporates the hydrological cycle (Palmer, 1965; Wells *et al.*, 2004), and the Normalized Difference Vegetation Index (NDVI), which relies on satellite imaging to quantify vegetation ‘greenness’ (Rouse Jr *et al.*, 1974). Each index has strengths and weaknesses (Zargar *et al.*, 2011), and all have been used for predicting drought risks, but they are more valuable for long-term appraisals. The utility of drought indices in analysing short-term biological impacts, particularly in animals capable of behavioural and physiological adjustments, remains uncertain. However, integrating drought indices with other environmental processes presents a promising research direction, as shown by a recent assessment proposed by Crausbay *et al.* (2024), which integrates drought indices with vegetation types, canopy cover, slope, time since fire and other environmental features, and develops region-specific management actions for decreasing further exposure. These actions include managing environmental water, restoring sites affected by deforestation and urbanization, and promoting ecosystem persistence under drought conditions (Mathwin *et al.*, 2021). Despite the enormous potential of such analyses, caution is required before implementing actions, especially those related to water supplies to landscapes, which might damage existing habitats or favour the accumulation of predators (Mathwin *et al.*, 2021).

The analytical context discussed here requires moving from quantifying environmental drying risk to calculating case-specific exposure risks to drought. This process is challenging, partially because hydrological variables such as rainfall, evapotranspiration and soil moisture content exhibit greater uncertainty than temperature trends, due to the stochastic nature of atmospheric processes (Wu *et al.*, 2024). Nonetheless, the expanding availability of independent hydrological models (Table 1) offers a timely opportunity to refine predictions of how reptiles and amphibians will respond to environmental water availability. Once ecologically relevant water variables are quantified, numerous approaches may integrate them explicitly with biological traits across different levels of organization—from molecules to phenotypes to communities. The interplay between exposure—typically to stressors such as water shortages, drought periods, or flooding—and biological response is key to understanding structural and functional consequences, providing indicators of sensitivity and vulnerability. The following section discusses sensitivity and vulnerability assessments, with a particular focus on the central theme of this review: hydroregulation.

### Linking exposure, sensitivity and vulnerability

The vocabulary of ecological climate change research has become complex, with many terms defining interrelated, yet different, concepts, including ‘Sensitivity’, ‘Vulnerability’, ‘Risk’, and ‘Resilience’. These terms have gained prominence across scientific and political discussion, as they are embedded in global agendas on sustainable development, disaster risk reduction, climate change and biodiversity loss (Williams *et al.*, 2008; Scholz *et al.*, 2012; Birkmann *et al.*, 2020). While we acknowledge this conceptual variability, our focus is on identifying the most relevant information for predicting future trends and anticipating species declines. To this end, various methodological tools and conceptual frameworks have been employed to assess how organisms and species respond to climate changes. However, quantifying responses is inherently complex, as environmental variability operates across all possible spatial and temporal scales, and science requires operationalization to specific cases. For instance, both exposure to climate change and species sensitivity to environmental shifts can differ dramatically within reptiles and amphibians. Linking exposure, sensitivity and vulnerability is a goal benefitted by advances in computational power, more sophisticated statistical models and large databases, which promote conceptual and disciplinary bridges. For example, connecting environmental changes not only with physiology and behaviour but also with conservation biology and ecosystem ecology (Cooke *et al.*, 2013; Madliger *et al.*, 2018). Methodological approaches have also evolved rapidly, and the most advanced tools explicitly link climate variables with organismal response by considering the underlying physiological and behavioural mechanisms that govern their survival and distribution (see ‘Assessing vulnerability: integrating exposure and sensitivity’ section).

Porter *et al.* (1973) and Tracy (1976) developed general microclimate models for quantifying the energy, heat and water budgets of organisms that have been revisited taking into consideration current computing power. Nowadays, it is possible to calculate microclimate at any location, and with fine temporal resolutions (reviewed in Meyer *et al.*, 2023; Kemppinen *et al.*, 2024). Some programmes even integrate microclimate (Table 1) with the calculated heat and water budgets of organisms (Kearney and Porter, 2020; Kearney and Enriquez-Urzelai, 2023) to estimate tolerance and distribution limits of organisms under real or any simulated climate (Kearney *et al.*, 2018; Cheng *et al.*, 2023). Relative to correlational models (Elith and Leathwick, 2009; Peterson *et al.*, 2011), these developments have added capacity for mechanistic predictions based on physiological limits of vulnerability to climate change (Riddell *et al.*, 2021; Briscoe *et al.*, 2023; Pottier *et al.*, 2025), bringing physiological data into the equation.

## Species Sensitivity Risk: Short-Term Impacts

Many acute and long-term responses to environmental drying are parallel to those triggered by other stressors (e.g. temperature, pollutants, food restriction), including altered metabolism, cardiovascular responses, growth, cellular oxidative stress, neuroendocrine pathways and gene expression. These common biomarkers are well documented in the literature, both in terms of methodology and interpretation (Bustin *et al.*, 2009; Ribou, 2016; Moretti *et al.*, 2017; Madliger *et al.*, 2018; Lighton, 2019). Here, we highlight some common responses to water stress, with a focus on water-specific responses and minimally invasive methods, outlined in Table 2. Most comparative studies compare arid-adapted and non-arid-adapted species, while experimental studies often involve subjecting animals to restricted water sources or increased environmental dryness.

**Table 2:** Example measurements to estimate an animal’s water loss/balance with definitions and calculations, and their interpretation for reptiles and amphibians

Name	Definition/calculation	Interpretation
EWL	Water loss through evaporative surfaces. Typically expressed as rate of water loss per unit time (g h^−1^), or resistance to water loss (s cm^−1^) Measurements of EWL can be whole-body, regional (e.g. ocular, dorsal, ventral, cloacal), exposed cutaneous surface area or respiratory.	Indicator of the animals’ risk of drying to the environment.
Water content	Whole-body mass: The amount of water in the animal. Typically expressed as percentage of whole mass or dry mass (%) in relation to standard (hydrated) body mass. Muscle: The amount of water in a sample of muscle tissue.	How much water is stored and available for the animal to use. Note that fat storage is another source of water through aerobic metabolism.
Blood biochemistry	Osmolality: Biomarker that measures the concentration of dissolved solutes in the blood. Typically expressed as milliosmoles per kilogramme of solvent (mosmol kg^−1^). Haematocrit: Proportion of blood volume occupied by red blood cells. Expressed as percentage of blood volume (%).	Indicator of dehydration status.
Water flux	Isotopic analysis of DLW, which traces the movement of water molecules between the organism and environment. Typically expressed as ml kg^−1^ day^−1^.	Estimate of daily water flux from free-ranging animals. Usually not suitable for semi-aquatic and aquatic species.
Water-seeking or conserving behaviour	Behaviours associated with seeking water (directional movement) and/or saving water (posture to diminish exposed body surface areas, shelter seeking, inactivity).	Indicator of behavioural focus on water balance.

### Genetic responses

**Figure 3:**
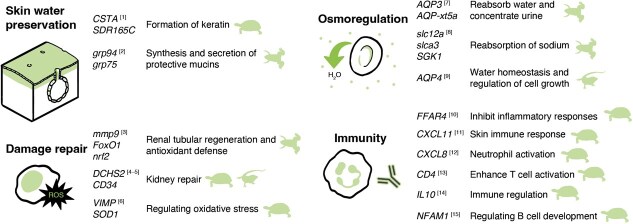
**Example genes identified in response to water stress**. Genes are grouped by the following functions: skin water preservation, osmoregulation, damage repair and immunity. Specific functions of the genes are described on the right side with the taxon in which the function has been demonstrated. Numbers inside the square bracket indicate the species and reference, *Gopherus agassizii*^[1,4,6,10–15]^ (Tollis *et al.*, 2017), *X. laevis*^[2,3]^ (Caine and Mclaughlin, 2013; Malik *et al.*, 2023), *Liolaemus fuscus*^[5,9]^ (Araya-Donoso *et al.*, 2022), *Xenopus tropicalis*^[7]^ (Shibata *et al.*, 2014) and *X. laevis/Bufo viridis/Fejervarya cancrivora*^[8]^ (Li *et al.*, 2022).

Genetic responses to environmental stress are broad across the literature. Here, we focus on three areas of hydroregulation: (1) the skin barrier, (2) water reabsorption and (3) cellular repair and immunity (Fig. 3). At the site of evaporation, the skin barrier of reptiles is regulated by the epidermal differentiation complex (EDC) gene cluster, which encodes proteins essential for keratinized cells in amniote skin. Among these, corneous beta-proteins (CBPs) genes are crucial for forming the outer layer of the skin, the stratum corneum (Holthaus *et al.*, 2024), while the *Loricrin* gene supports alpha-keratinization in lizard epidermis (Holthaus and Eckhart, 2024). Under arid conditions, the upregulated expression of CBPs and *Loricrin* increases the thickness and strength of the stratum corneum, enhancing resistance to dehydration. Comparative genomics between the desert tortoise (*Gopherus agassizii*) and the temperate aquatic western painted turtle (*Chrysemys picta bellii*) have identified multiple positively selected genes associated with drought resistance. These genes include *CSTA* and *SDR16C5* (Fig. 3)*,* which are involved in keratin formation and lipid-based waterproofing, respectively, and are likely positively selected in arid environments (Tollis *et al.*, 2017). In contrast, amphibians rely on mucous secretions to minimize water loss, along with other functional roles. For example, conserved genes such as *grp94* and *grp75* (related to glucose-regulated proteins), which are widely present across taxonomic groups, exhibit dehydration-induced upregulation in *Xenopus laevis*, promoting the synthesis and secretion of protective glycoproteins to reduce water loss while preserving skin moisture (Malik *et al.*, 2023).

Animals adapted to arid conditions exhibit a strong capacity for water reabsorption, enabling them to produce highly concentrated urine while maintaining salt balance. Several genes are essential for cellular water reabsorption, particularly those encoding membrane proteins forming water channels such as aquaporins (AQP). AQP genes, widely present in amphibians and reptiles (Fig. 3), and the proteins they encode are crucial for osmoregulation, including transcellular water and solute transport under both low and high osmotic stress (Suzuki and Tanaka, 2009; Shibata *et al.*, 2014; Chen *et al.*, 2019; Wu *et al.*, 2019; Lemenager *et al.*, 2022). On the other end of osmoregulation, water balance can also be regulated by moving chloride along with sodium or potassium, creating osmotic gradients between cells and their surrounding environment, which drive water reabsorption through osmosis. This process requires ion transport proteins, including those in the Solute Carrier 12 (*Slc12*) family of cation-coupled chloride cotransporters (Fig. 3) (Motoshima *et al.*, 2023). The proteins encoded by these genes facilitate sodium reabsorption in renal structures like the distal convoluted tubule and thick ascending limb, generating a hyperosmotic environment that drives water reabsorption via osmosis (Marra *et al.*, 2012). While the *Slc12* family is widely conserved across vertebrates, *Slc12a1* exhibits significant upregulation in desert-adapted species, suggesting its critical role in arid environmental osmoregulation (Marra *et al.*, 2012).

The kidney is the epitomic organ in water homeostasis, filtering waste while regulating water and ion balance, making it particularly vulnerable to dehydration stress. Thus, impaired kidney function figures among the various forms of stress imposed by chronic water deprivation in amphibians and reptiles. Systems of protection have evolved in lizards and turtles adapted to arid environments, a condition thought to positively select genes associated with kidney repair, such as *DCHS2,* related to cell adhesion, and *CD34*, linked to vascular repair, both of which harbour functional mutations in desert-dwelling species (Fig. 3) (Tollis *et al.*, 2017; Araya-Donoso *et al.*, 2022). Another physiological challenge caused by dehydration is disruption of cellular homeostasis, leading to oxidative stress in the accumulation of reactive oxygen species, causing tissue damage (Dupoué *et al.*, 2020c; Ritchie and Friesen, 2022). However, amphibians and reptiles adapted to arid environments activate antioxidant defence mechanisms to mitigate dehydration-induced oxidative stress (Moreira *et al.*, 2020; de Amaral *et al.*, 2024). For instance, numerous antioxidant and detoxification genes are regulated by the transcription factor erythroid 2-related factor 2 (*Nrf2*), a key regulator of oxidative stress responses under elevated reactive oxygen species levels, and are widely conserved across species, with its protein content significantly enhanced in *X. laevis* during dehydration (Fig. 3) (Malik and Storey, 2009). With declining genetic sequencing costs and expanding analytical capacity, the coming years hold promise for advancing our understanding of the genetic responses of amphibians and reptiles to hydric stress.

### Hormonal responses

One of the most well-documented effects of pond drying are neuroendocrine responses, which have been extensively studied in amphibians. Water-dependent tadpoles can adjust their developmental rate and, therefore the timing of metamorphosis in response to environmental changes (Newman, 1988; Lai *et al.*, 2002; Benard, 2004; Wu and Kam, 2009; Higginson and Ruxton, 2010; Thompson and Popescu, 2021; Sinai *et al.*, 2022). These environmental cues stimulate the central nervous system, activating the hypothalamo–pituitary–interrenal/adrenal axis to initiate survival mechanisms. During pond drying, the hypothalamus increases the production of a corticotropin-releasing hormone (CRH), stimulating the release of the adrenocorticotropic hormone (ACTH) and thyroid-stimulating hormone (TSH) from the pituitary. This, in turn, activates the thyroid and interrenal glands, elevating thyroid hormones (THs) and corticosterone (CORT) in the bloodstream, which help manage stress, metabolism and developmental transitions (Kikuyama *et al.*, 1993; Denver, 1997; Kirschman *et al.*, 2017; Ruthsatz *et al.*, 2020). Increased hormone production accelerates metamorphosis, thus shortening the larval period and improving survival as aquatic habitats shrink (Denver, 2013). This hormonal plasticity highlights the resilience of some amphibians, enabling them to cope with environmental fluctuations and complete their life cycle under harsh conditions. Some hormones, such as CORT, are released into surrounding waters through various mechanisms (e.g. secretion and diffusion), and remain stable long enough to be quantified. Therefore, it can be measured non-invasively from water samples, allowing researchers and managers to monitor stress in both laboratory and field settings (Ruthsatz *et al.*, 2023a; Ruthsatz *et al.*, 2023b), However, waterborne and plasma CORT levels may vary across species (Millikin *et al.*, 2019) and depend on environmental contexts (Mausbach *et al.*, 2022).

In terrestrial amphibians and reptiles, acute dehydration triggers fluid balance responses via mineralocorticoid hormones such as aldosterone and peptides including arginine vasopressin and angiotensin, all of which play key roles in water metabolism, helping organisms retain water and maintain circulatory stability under dehydration stress (McCormick and Bradshaw, 2006; Uchiyama and Konno, 2006; Dantzler and Bradshaw, 2008; Hillman *et al.*, 2009). CORT also contributes to hydroregulation through its mineralocorticoid actions (McCormick and Bradshaw, 2006; Dupoué *et al.*, 2016; Brusch *et al.*, 2020), though it may not consistently correlate with plasma osmolality within a species (Dezetter *et al.*, 2022b). Elevated CORT may mobilize energy reserves via muscle catabolism, reallocating bound water to maintain hydration (Brusch *et al.*, 2018; Dezetter *et al.*, 2021). These hormones also regulate other interrelated processes, including energy metabolism, reproduction, social behaviour and thermoregulation (Ladyman *et al.*, 2006; Bleu *et al.*, 2013; Carsia *et al.*, 2023; Crino *et al.*, 2024). Therefore, when evaluating hormonal responses to dehydration, researchers should consider these overlapping physiological functions and assess additional traits linked to hydration and water balance.

### Other physiological responses

Physiological responses and regulation to acute water stress include osmoregulation, cardiovascular function, metabolism, immunity and the renin–angiotensin–aldosterone system (as mentioned above) are well documented in the literature for amphibians (Feder and Burggren, 1992; Hillman *et al.*, 2009) and reptiles (Pough and Gans, 1982; Dantzler and Bradshaw, 2008; Bradshaw, 2012). Here, we focus on traits commonly measured with conservation relevance, emphasizing minimally invasive protocols (Table 2). These can be broadly classified into (1) water loss through evaporation, (2) hydration state and (3) daily water flux.

Evaporative water loss (EWL) is of considerable interest because it responds immediately to low air humidity (Mautz, 1980; Hillman *et al.*, 2009). Evaporation depends on both biophysics (Foley and Spotila, 1978; Campbell and Norman, 2000) and hydration state (Anderson *et al.*, 2017; Senzano and Andrade, 2018; Weaver *et al.*, 2022), but also on physiology, so that the rate of water loss tends to be lower in comparable counterparts from more arid environments, across populations and species (Bentley and Schmidt-Nielsen, 1966; Roberts and Lillywhite, 1983; Cox and Cox, 2015; Salazar and Miles, 2024). EWL mainly occurs through respiratory and cutaneous pathways, with some influence from ocular and cloacal pathways (Fig. 1) (Hillman *et al.*, 2009; Pirtle *et al.*, 2019), and the combination of these pathways (total EWL, or TEWL) can be measured simply by the mass loss of the animal (or mass gain of a desiccant) over time, or by respirometry methods (Hillman *et al.*, 2009; Lighton and Halsey, 2011). Physical models (Senzano *et al.*, 2022) or mathematical approaches (Riddell *et al.*, 2017), which can be used to quantify EWL, but special consideration of boundary layers is required. These thin layers of fluid (air or water) that form at the interface between an organism’s body and its surrounding environment, usually affect heat, water and gas exchange. Experimentally, respiratory and cutaneous EWL (REWL and CEWL, respectively) can be distinguished by placing a mask with separate airflow for the lungs and skin (Withers, 1977; Senzano and Andrade, 2018) or by using an impervious membrane to isolate body regions (Dmiel, 2001). CEWL can also be measured directly with an evaporimeter in a flux chamber (Lillywhite *et al.*, 2009; Tingley *et al.*, 2012; Oufiero and Van Sant, 2018), a method that has the added benefit of focusing on specific body regions (Weaver *et al.*, 2022; Weaver *et al.*, 2023). For broader comparisons, global databases of EWL for frogs and squamates are available to statistically tease out environmental and phylogenetic drivers of EWL (Cox and Cox, 2015; Le Galliard *et al.*, 2021b; Wu *et al.*, 2024a). However, EWL datasets for other herpetofauna groups, such as Crocodilia, Testudines, Caudata and Gymnophiona, remain relatively scarce compared to those for frogs and squamates.

Animals in dry environments survive by efficiently storing water, producing metabolic water, mobilizing water from tissues and tolerating low body water content (Cloudsley-Thompson and Cloudsley-Thompson, 1999; Hillman *et al.*, 2009; Lillywhite, 2016). The water content in the body of an animal is expressed as a percentage of whole mass or dry mass and has historically been measured by fully desiccating specimens (Thorson, 1955; Pough *et al.*, 1983; Taigen *et al.*, 1984). Amphibians typically have 77–83% water content by body weight (Hillman *et al.*, 2009), while reptiles range from 63 to 74% (Thorson, 1968). Shifts in body water allocation may support water balance when facing dehydration, and the nature of such pathways varies across lineages. For example, amphibians can absorb water from their bladders (Sawyer and Schisgall, 1956; Schmuck and Linsenmair, 1997; Suzuki *et al.*, 2015), while snakes and lizards rely on CORT-mediated muscle catabolism to release water originally associated with proteins (bound water), and may obtain water as a byproduct of lipid metabolism (Brusch *et al.*, 2018; Dezetter *et al.*, 2021). Internal water mobilization can be tracked through changes in blood nutrients, proteins, triglycerides, uric acid, mineralocorticoid hormones as well as transcriptome and proteome changes in blood and tissue samples (Suzuki *et al.*, 2015; Brusch *et al.*, 2018). Given recently established pathways for muscle catabolism in snakes and lizards, non-invasive methods, such as specimeters, now quantify muscle changes as proxies for water balance in reptiles (Lourdais *et al.*, 2005; Dezetter *et al.*, 2021). In amphibian research, a method to assess hydration states involves measuring the body mass of field-captured individuals, then allowing them to fully rehydrate in a field lab, and recording the subsequent mass. The difference between the initial field mass and the fully hydrated mass indicates the degree of dehydration experienced in their environment. For instance, a study on tropical frog species found that hydration behaviours and voluntary tolerance of dehydration varied with habitat use, even among closely related species within the same family (Tracy *et al.*, 2014). This approach provides insights into species-specific water balance strategies and their adaptability to varying environmental conditions.

Blood biochemistry parameters, such as plasma osmolality and haematocrit, serve as indirect measures of hydration status (Table 1) in three dominant contexts, field studies (Capehart *et al.*, 2016; Moeller *et al.*, 2017; Brischoux and Cheron, 2019; Weaver *et al.*, 2024), laboratory experiments (Dupoué *et al.*, 2017; Wu *et al.*, 2017; Dezetter *et al.*, 2022b; Chabaud *et al.*, 2023) and veterinary applications (Perry *et al.*, 2020; Cameron *et al.*, 2024). Plasma osmolality is best measured using vapour pressure or freezing-point depression osmometers (Nevarez *et al.*, 2012; Wright *et al.*, 2013; Buchmiller *et al.*, 2024), as formulas based on solute concentrations often show poor agreement with direct measurements (Dallwig *et al.*, 2010; Nevarez *et al.*, 2012; Perry *et al.*, 2020). Haematocrit is determined by centrifuging blood in microcapillary tubes, and it is frequently used as a proxy for hydration status, although it responds to multiple influencing factors such as blood oxygen-carrying capacity (Brischoux *et al.*, 2011; Lourdais *et al.*, 2014; Bodensteiner *et al.*, 2021) and does not consistently correlate with plasma osmolality (Dupoue *et al.*, 2015; Dezetter *et al.*, 2021). Therefore, interpreting haematocrit changes as indicators of hydration requires caution, considering additional factors affecting blood viscosity and oxygen transport. Ecologically relevant osmolality measurements should incorporate species-specific normosmotic values, tolerance to variation, temporal dynamics of osmolality shifts and threshold effects on physiological and behavioural water balance regulation (Dessauer, 1970). Notably, species from xeric environments tolerate greater osmolality fluctuations than those from mesic habitats, such as extreme water deprivation and excess water, underscoring the importance of species-specific considerations in hydration studies (Nagy and Medica, 1986; Brusch and DeNardo, 2017).

Whole-animal water flux, encompassing influx, storage and efflux (Fig. 1), can be quantified using doubly labelled water (DLW), which estimates field metabolic rate and water flux over extended periods (Table 1) (Nagy, 1989). DLW has been widely applied to measure daily water flux in reptiles across diverse field conditions (Beaupre, 1996; Christian *et al.*, 1999; Christian *et al.*, 2007; Roe *et al.*, 2008; Harden *et al.*, 2014). For instance, velvet geckos from arid zones exhibit lower water flux rates year-round compared to those in wet tropical regions, reflecting adaptive water conservation strategies (Christian *et al.*, 1998). The ability to estimate both field metabolic rate and water flux makes DLW a powerful tool for field-based physiological research. However, certain assumptions in DLW-derived metabolic rates can introduce measurement errors (Nagy, 1980). For example, high humidity can overestimate metabolic rates due to excessive water vapour exchange through cutaneous and respiratory surfaces, whereas total water flux aligns more reliably with gravimetric estimates of TEWL (Anderson *et al.*, 2003). Additionally, DLW is unsuitable for species with high water flux, such as semi-aquatic reptiles, because rapid water turnover depletes isotopes too quickly, preventing accurate measurements (Booth, 2002; Jones *et al.*, 2009). The method has limited use for amphibians due to their high water fluxes, but if the primary objective is to assess water turnover, the method could be applied to more terrestrial amphibian species (Christian and Green, 1994). This would offer valuable insights into the water cost of activity and dispersal under field conditions.

### Behavioural responses

Terrestrial amphibians and reptiles employ diverse behavioural strategies to regulate water balance, which can be broadly classified into (1) water-conserving behaviours, (2) water-seeking behaviours and (3) moisture-harvesting behaviours. These strategies mitigate EWL, optimize hydration and enhance survival in desiccating environments. Water-conserving behaviours are those minimizing exposure to drying conditions. When avoidance of dehydration is no longer viable, animals may engage in water-seeking behaviours, actively locating and consuming water to restore hydration, or actively collecting and absorbing water from their surroundings. A universal water-conserving behaviour involves reducing or shifting diel activity and selecting microhabitats that provide moisture, such as burrows, and this type of behaviour has been observed in the field (Daltry *et al.*, 1998; Davis and DeNardo, 2010; Kearney *et al.*, 2018; Moore *et al.*, 2018) and experimentally (Navas *et al.*, 2002; Székely *et al.*, 2018; Rozen-Rechels *et al.*, 2020; Dezetter *et al.*, 2023). Also, water-conserving behaviours relate to body temperatures, which usually enhance rates of water loss (Tracy *et al.*, 2008; Dupoué *et al.*, 2015; Lourdais *et al.*, 2017). Therefore, shifts in thermoregulatory behaviour, including thermal depression, can contribute to water-conserving strategies (Malvin and Wood, 1991; Ladyman and Bradshaw, 2003; Anderson and Andrade, 2017; Le Galliard *et al.*, 2021a; Camacho *et al.*, 2023). Although the interplay between water and heat budgets complicates the disentangling of hydroregulation and thermoregulation mechanisms (da Silveira Scarpellini *et al.*, 2015; Pintor *et al.*, 2016; Rozen-Rechels *et al.*, 2019), recent modelling approaches considering both joint mechanisms and microclimatic data are improving our understanding of behavioural responses to drying and heating (Kearney *et al.*, 2018; Moore *et al.*, 2018; Encarnación-Luévano *et al.*, 2021). By strictly controlling for temperature, experimental studies have demonstrated hydroregulation behaviours through the active selection of moister microclimate in both wet-skinned amphibians (Mitchell and Bergmann, 2016) and dry-skinned reptiles (Dezetter *et al.*, 2023). This behaviour mitigates the acute effects of desiccating conditions. These findings suggest that both resistance to water loss and hydric performance response curves may influence the timing of behavioural responses to drying in reptiles and amphibians.

Some animals can reduce water loss by modifying body posture and preferring those that reduce the exposed area to the environment (Table 2). Placing limbs against the body and using skin folds to cover ventral surfaces against the substrate, as in Anura (Pough *et al.*, 1983; Tattersall *et al.*, 2006) or coiling in Caudata (Cohen, 1952) greatly reduce TEWL (Spotila and Berman, 1976). In addition to postural changes, several species of arboreal frogs use limbs to spread waxy films over their body surfaces during dry seasons or produce cocoons to reduce CEWL (Lillywhite, 2006). By manipulating the hydration state via moisture gradients and assessing postural adjustments, experimental studies can examine the determinants of these behaviours and their benefits for maintaining hydration (Navas *et al.*, 2002; Mitchell and Bergmann, 2016). In reptiles, behaviours such as coiling in snakes or adopting tucked-in postures in lizards may also confer water-saving benefits. However, this aspect has received comparatively limited attention, mostly restricted to studies on egg-brooding behaviour in snakes, where subtle postural shifts can reduce egg surface exposure and limit water loss from the egg clutch (Lourdais *et al.*, 2007; Stahlschmidt *et al.*, 2008; Stahlschmidt and DeNardo, 2010). Finally, the simple closure of eyes can help reduce water loss through the permeable eye membrane in lizards (Pirtle *et al.*, 2019).

When avoiding and restricting drying is no longer possible, reptiles and at least some amphibians will seek water to restore their hydration state (Table 2). Experimental systems called ‘olfactometers’ designed by Grubb (1973), and follow-up studies with maze designs, have demonstrated that frogs and lizards can detect and locate free-standing water via olfactory cues (Navas *et al.*, 2002; Madelaire *et al.*, 2020; Ouellet *et al.*, 2020; Lorrain-Soligon *et al.*, 2022; Northrop, 2024). However, generalizing is not possible for amphibians. Finding generic water for hydration and finding specific waters for reproduction seem to be independent processes, and both have been identified in some species. For example, telemetric studies show that poison frogs rely on odour cues from stagnant water to find new breeding pools (Serrano-Rojas and Pašukonis, 2021). However, this ability varies across species (Reshetnikov, 1998; Maia, 2014) and may relate to drying tolerance and habitat aridity (Cruz-Piedrahita *et al.*, 2018; Galindo *et al.*, 2024). Particularly, some anuran species rely on structured water search strategies, while others find water by erratic exploration (Maia, 2014). Finally, drinking matters for some species only as others will rely on a specialized, richly vascularized region of the pelvic skin (Willumsen *et al.*, 2007). It has also been reported for snakes subjected to field experimental dehydration or rehydration after capture (Brischoux *et al.*, 2017; Dezetter *et al.*, 2022b) and is triggered by physiological thresholds such as hydration status (Sandfoss and Lillywhite, 2019; Edwards *et al.*, 2021). Quantifying water-seeking behaviour (e.g. time to find water source) should be of consideration for habitat restoration managers when optimizing water resources for herpetofauna to persist and flourish in a given habitat (Mathwin *et al.*, 2021).

Moisture and rain-harvesting behaviours are also observed in reptiles (Sherbrooke, 1993; Joel *et al.*, 2017). These behaviours include snakes coiling and flattening their bodies, lizards flattening their bodies and both lizards and tortoises raising their abdomen and lowering their heads and tails (Repp and Schuett, 2008; Glaudas, 2009; Yenmiş *et al.*, 2024). Similarly, some postural adjustments in amphibians can facilitate moisture and water uptake through the skin, particularly through the pelvic patch (McClanahan Jr and Baldwin, 1969; Bentley and Main, 1972; Hillyard *et al.*, 1998; Word and Hillman, 2005; Tracy *et al.*, 2011). Overall, water-searching behaviours and related adaptations are critical for understanding species sensitivity and resilience to aridification. For instance, invasive frogs at the forefront of their invasion show distinct water-searching tendencies, with stress differentially affecting this behaviour (Madelaire *et al.*, 2020).

### Life history responses

Water stress can impact life history by influencing (1) growth and the rate of development, (2) body size and (3) reproduction. Animals can adjust their rate of development under different environmental conditions (see hormonal control under the ‘Hormonal responses’ section). This developmental plasticity can be either adaptive or maladaptive, depending on whether the developmental environment matches the conditions an individual experiences later in life (Monaghan, 2008; Beaman *et al.*, 2016). For the larval stages of amphibians, reduced water availability, such as pond drying, can accelerate larval development, leading to smaller body sizes or incomplete metamorphosis due to resource constraints, crowding, poorer water quality and increased predation risk (Márquez-García *et al.*, 2010; Gomez-Mestre *et al.*, 2013; Albecker *et al.*, 2023). Some species, however, do not show changes in developmental rate nor exhibit delayed larval development under drying conditions (Richter-Boix *et al.*, 2011), promoting the importance of species-specific responses. It is also clear that developmental plasticity to pond drying can have carryover effects on post-metamorph individuals and adults. Under pond drying conditions, metamorphs have lower thermal tolerance, are less exploratory and more stressed and have lower jumping performance and lower immunity (Gervasi and Foufopoulos, 2008; Crespi and Warne, 2013; Charbonnier *et al.*, 2018; Brannelly *et al.*, 2019; Ohmer *et al.*, 2023; Nolan *et al.*, 2025; Wu *et al.*, 2025). Size is particularly important because larger individuals are associated with increased survival rate, performance (Cabrera-Guzmán *et al.*, 2013) and lower risk to disease progression for the same pathogen load (Brannelly *et al.*, 2018; Wu *et al.*, 2018).

Water availability also plays a critical role in the reproductive success of egg-laying reptiles, influencing both egg survival and offspring development. For species that lay eggs on land, eggshell thickness and composition are key determinants of desiccation risk. Flexible-shelled eggs, which lack or have minimal calcareous layers (most squamates and some chelonians), are more porous and susceptible to water loss compared to rigid-shelled eggs with a well-developed calcareous layer (crocodilians, some chelonians, and a few squamates) (Legendre *et al.*, 2022). A meta-analysis showed that substrate moisture had a small but significant effect on hatchling length and mass for reptiles, as well as on sex ratios specifically for chelonians, but not on incubation duration (Bell *et al.*, 2025). However, this meta-analysis did not consider differences in eggshell type due to phylogenetic biases in categorizing shell type. Species that nest in arid environments tend to have highly absorbent and thicker shells, suggesting that species with flexible eggshells may be more vulnerable to environmental drying (D'Alba *et al.*, 2021; Debruyn *et al.*, 2023). At the other extreme, excessive moisture can also be detrimental, leading to reduced oxygen availability in nests, lower hatchling success, and high embryo mortality (Marco and Díaz-Paniagua, 2008; Gatto and Reina, 2022; Warner *et al.*, 2023). However, some tropical species are adapted to excessive moisture events such as flooding, through arrested development of the eggs (Kennett *et al.*, 1993; Seymour *et al.*, 1997), which therefore highlights species-specific assessments of extreme moisture events on hatching success. These findings underscore that water management in nesting habitats of reptiles is as crucial for egg and juvenile survival as it is for adult life history strategies in response to environmental dryness.

Drying stress during reproduction and early life can impact reproductive output and offspring phenotypes in reptiles (Dupoué *et al.*, 2018; Dupoué *et al.*, 2020b; Dezetter *et al.*, 2021). Successful reproduction requires substantial water investment, particularly during gravidity in oviparous reptiles (Brusch *et al.*, 2019; Dupoué *et al.*, 2020a,), and even more so in viviparous species, where pregnant females experience increasing hydration demands as embryos develop *in utero* (Dupoue *et al.*, 2015; Lourdais *et al.*, 2015; Lourdais *et al.*, 2017). To reduce water loss, gravid females may adjust their behaviour, seeking moister microhabitats (Lourdais *et al.*, 2017). However, under limited water availability, they face a trade-off between self-maintenance and offspring investment, often prioritizing embryonic water allocation at their own physiological expense (Dupoue *et al.*, 2015; Dupoué *et al.*, 2020a; Dezetter *et al.*, 2021). Maternal dehydration can have severe reproductive consequences, including follicular resorption at early stages (Capehart *et al.*, 2016; Zani and Stein, 2018), reduced investment in eggs, and thinner eggshells with modified immune function (Brusch *et al.*, 2019). In later stages, maternal water deprivation increases embryonic mortality (Dezetter *et al.*, 2021), potentially contributing to drought-driven population decline (Madsen *et al.*, 2023). These demographic costs may be exacerbated by fecundity trade-offs, as larger females carrying more embryos experience greater physiological stress (Dupoue *et al.*, 2015; Lourdais *et al.*, 2015; Dezetter *et al.*, 2021). However, the generality of maternal–offspring water trade-offs remains uncertain, as some species, such as *Anolis sagrei*, exhibit no observable effects of maternal dehydration on fecundity, egg size or egg hydration (Wayne *et al.*, 2025). While most studies have focused on dehydration to explore the hydric costs of reproduction, hyperhydration may also pose distinct challenges, particularly when reproductive life stages depend on aquatic or more humid habitats. Migration from terrestrial habitats to breeding ponds during reproduction in male toads, for instance, can result in hyperhydration challenges (Brischoux and Cheron, 2019). Yet, far less is known about the effects of excess water stress on reproduction and developmental rate.

## Species Sensitivity Risk: Long-Term Impacts

Beyond the immediate effects of drying, animals must also cope with longer periods of desiccation for populations to survive. Understanding the long-term implications of water limitation requires an integrative approach that incorporates adaptation, plasticity and demographic shifts through experimental and field-based studies. This section explores two key aspects of long-term water deficit impacts: (1) the role of heritability and plasticity in hydroregulation traits and (2) the influence of water availability on body size evolution.

### Heritability and plasticity of hydroregulation traits

Repeatability and heritability experiments are key to determining whether hydroregulation traits are targets of natural selection. Although often labour-intensive, repeatability measures the consistency of a trait within individuals under similar physiological conditions, whereas heritability assesses its genetic transmission across generations (Wolak *et al.*, 2012). However, our understanding of the repeatability and heritability of hydroregulation traits remains limited. Empirical studies provide some insights into the genetic and phenotypic basis of these traits. In reptiles, significant repeatability of EWL in *Sceloporus consobrinus* (Oufiero and Van Sant, 2018), and moderate heritability of desiccation tolerance in *Lampropholis* skinks have been observed (Martins *et al.*, 2019). In amphibians, covariance between thermal traits and skin resistance to water loss has been observed in *Plethodon metcalfi* (McTernan and Sears, 2022). While these studies suggest that hydroregulation traits exhibit some degree of plasticity, especially in reptiles, large-scale assessments of frog populations across natural climatic gradients indicate low variation in EWL rates, suggesting potential constraints on plasticity for amphibians (Davies *et al.*, 2015; Bovo *et al.*, 2023). These findings emphasize the need for broader geographic and taxonomic coverage (White *et al.*, 2021; Herrando-Pérez *et al.*, 2023) to clarify the magnitude of variation in hydroregulation traits and how they scale over time to shape long-term responses to environmental change.

Acclimation, a laboratory-based phenomenon resulting from the deliberate alteration of a single environmental parameter, is among the most studied types of plasticity. Acclimation refers to the ability of organisms to adjust their phenotype in response to environmental stressor(s), another key indicator of organisms’ capacity to survive in changing climates, including water-scarce environments. Our understanding of potential adjustments in hydroregulation traits is limited compared to thermal physiological counterpart traits (Seebacher *et al.*, 2015; Bovo *et al.*, 2018), although some experimental studies have explored how organisms modify hydroregulation in response to thermal acclimation (Davies *et al.*, 2015). For example, thermal acclimation during development can lead to changes in TEWL that persist until adulthood in snakes (Dezetter *et al.*, 2022a), while the TEWL of lizards decreased in response to warmer temperatures (Vicenzi *et al.*, 2021). Riddell *et al.* (2019) highlighted that temperature is an important cue for developing a desiccation-resistant phenotype, by regulating water loss through the regression and regeneration of capillary beds in the skin. The growing literature on disentangling the differences in acclimation effects of temperature and drying exposure on hydroregulation provides a promising area for understanding long-term water restrictions or simulated drying to assess the plasticity of these traits across different species (Kobayashi *et al.*, 1983; Kattan and Lillywhite, 1989; Moen *et al.*, 2005; Riddell *et al.*, 2018a; Rozen-Rechels *et al.*, 2020a; Weaver *et al.*, 2023). Acclimatization, a response within an organism’s lifetime (days or weeks) to multiple stressors simultaneously (e.g. temperature, humidity, photoperiod) in its natural environment, should also be considered for informing a species’ long-term risk to climate change, because an organism’s physiology can differ by, for instance, seasonality, which may represent an important type of plasticity to cope with climate changes (Dubiner *et al.*, 2023; Day *et al.*, 2025).

### Water availability and body size evolution

One notable potential long-term effect of changes in precipitation is altered body size (Gouveia and Correia, 2016; Guo *et al.*, 2019; Pincheira-Donoso *et al.*, 2019). Two contrasting mechanisms have been proposed to explain this relationship: (1) the ‘resource hypothesis’, where higher rainfall boosts primary productivity, supporting larger individuals due to greater food availability (Rosenzweig, 1968), and the ‘water conservation hypothesis’, where arid environments favour larger individuals because lower surface-area-to-volume ratios reduce EWL relative to smaller individuals (Nevo, 1973; Gouveia and Correia, 2016). Evidence from reptile communities supports the resource hypothesis, with some species increasing in size as precipitation rises (Stanley *et al.*, 2020). However, amphibians show a more complex pattern, with larger body associated with higher precipitation in cooler climates but also with lower precipitation in warmer regions, possibly indicating a transition from resource-driven to desiccation resistance-driven selection (Sheridan *et al.*, 2022). Despite these findings, body size responses to climatic water balance remain debated (Servino *et al.*, 2022). In contrast with reptiles, the permeable skin of amphibians makes them particularly vulnerable to desiccation. This key difference may contribute to diverging size trends between reptile and amphibian communities in response to water availability. To clarify these patterns across sites, long-term body size monitoring in conjunction with environmental data—including analyses of museum specimens with historical climate records—can help elucidate the drivers of body size evolution. Further research is needed to assess long-term changes in skin permeability and their potential correlation with body size variation.

## Assessing Vulnerability: Integrating Exposure and Sensitivity

### Vulnerability indices and organismal traits

A number of vulnerability indices of physiological stress, extinction risk, activity time constraints, habitat suitability or range shifts have been proposed depending on the question of interest (Deutsch *et al.*, 2008; Kearney and Porter, 2009; Sinervo *et al.*, 2010; Lertzman-Lepofsky *et al.*, 2020; Souza *et al.*, 2023). These indices are projected across space and time and are based on the experimental estimation of fitness-related traits. Some indices relate environmental variables with physiological thresholds (e.g. desiccation tolerance, performance curves; Greenberg and Palen, 2021; Anderson *et al.*, 2023), whereas more complex counterparts are based on biophysical models designed to reflect energy and water exchanges between animals and their microclimatic environments (Kearney *et al.*, 2013; Kearney *et al.*, 2018; Briscoe *et al.*, 2023). Importantly, thermal biology information characterizes most indexes (Taylor *et al.*, 2020) despite the high relevance of hydroregulation for water-sensitive groups such as amphibians (Lertzman-Lepofsky *et al.*, 2020; Greenberg and Palen, 2021; Wu *et al.*, 2024a).

Models and indexes have been used to predict biological constraints on fitness, using as proxies development, growth, activity, reproduction and survival (Sinervo *et al.*, 2010; Kearney *et al.*, 2018). This is because fitness-related traits are key to informing vulnerability to a given source of physiological stress. Yet disagreements exist on whether traits and what traits are good predictors for informing causal links of environmental changes on populations and species (Calosi *et al.*, 2008; Beissinger and Riddell, 2021). For example, common modelling variables related to hydroregulation include hydration level, rates of water loss, measures of water acquisition (seeking out water sources or specific microhabitats that enhance water uptake or maintenance) and the concentration of body fluids (Table 2). Hydroregulation traits are integrated with various functions related to gas exchange, energetics, thermoregulation and reproduction as previously highlighted in section ‘Species sensitivity risk: short-term impacts’. Thus, and according to the physiology of the target groups, models exclusively based on hydroregulation may under- or overestimate vulnerability to climate change (Riddell *et al.*, 2018a; Rozen-Rechels *et al.*, 2019). The use of multiple physiological thresholds such as thermal tolerance, reproduction and growth with hydroregulation through experimental manipulation of environmental stressors or inputting appropriate parameters to mechanistic models will provide more holistic estimations of vulnerability to climate change.

### Challenges in predicting vulnerability

Predicting vulnerability to environmental stressors and how this may scale up to population- or species-level responses remains a key challenge for the conservation of biodiversity (Bovo *et al.*, 2018). Practical limitations include characterizing with appropriate data species-specific microclimates, both temporally and spatially (Briscoe *et al.*, 2023). These limitations extend to single-population estimates, and the sometimes-related use of an average value to represent a whole species. The validity of such approaches is context-specific, but they may not reflect across-population variation in sensitivity to thermal variation (Herrando-Pérez *et al.*, 2019; Senior *et al.*, 2019; Bovo *et al.*, 2023) and/or drying as well as population plasticity/adaptation to drying condition. The same principle applies to studies using closely related species to represent threatened counterparts (Reemeyer *et al.*, 2024). Mechanistic models that embrace population variability and plasticity in response to environmental drying will allow more explicit predictions of vulnerability across a species range (see ‘Future directions’). Validating these predictions is essential, particularly for models that estimate survival, reproduction and activity, which should be tested against observational data to ensure accuracy. Natural history observations (Greene, 2005) and large-scale longitudinal field and laboratory studies, particularly when there are geographically biased data (White *et al.*, 2021; Herrando-Pérez *et al.*, 2023), can help validate mechanistic models when predicting biological impacts of climate change across a species range or communities (Kearney *et al.*, 2018; Enriquez-Urzelai *et al.*, 2019; Riddell *et al.*, 2019; Briscoe *et al.*, 2023).

## Future Directions

### Linking gene expressions to functional changes in response to environmental drying

Whole-genome sequencing is becoming increasingly affordable and accessible for researchers and conservation biologists (Theissinger *et al.*, 2023; Hogg, 2024). As we previously highlighted, several genes have been identified that are linked to an animal’s hydroregulation. Understanding how the expression of these genes translates into functional changes in an animal’s water balance is key to uncovering the genetic mechanisms underlying plasticity in response to environmental drying (Somero, 2010). Riddell *et al.* (2019) identified, in salamanders, >500 genes in response to different temperature and VPD acclimation. Network analysis of these genes revealed suites of gene networks associated with the plasticity of skin resistance and the regulation of skin blood vessel growth. For example, the expression of ‘hydroperoxide isomerase’ (*ALOXE3*), a gene involved in regulating transepidermal water loss, was highlighted. This study underscores an important research direction for identifying which genes are targets of selection when inferring the adaptive potential of species to warming and drying environments. Epigenome-wide association studies represent a promising approach for establishing causal relationships between changes in the epigenome and phenotypic plasticity (Fanter *et al.*, 2022).

### Inter- and transgenerational plasticity in response to environmental drying

Parental environments can shape offspring phenotype via epigenetic mechanisms such as DNA methylation, histone modifications and non-coding RNAs (Galloway and Etterson, 2007; Beaman *et al.*, 2016; Loughland *et al.*, 2021; Husby, 2022). Intergenerational and transgenerational plasticity, which describe epigenetic inheritance across one or multiple generations, could buffer populations against environmental change, particularly if parental and offspring environments match (Shama and Wegner, 2014; Pettersen *et al.*, 2024). However, despite growing interest in these mechanisms, few studies have explored these processes in amphibians and reptiles. One promising research direction is to experimentally test whether epigenetic modifications induced by water stress persist across generations and whether they enhance desiccation resistance. This could involve controlled desiccation experiments, tracking epigenetic changes and hydroregulation traits across multiple generations (Dupoue *et al.*, 2015; Dupoué *et al.*, 2018,), or comparing populations from environments with different hydric regimes to assess whether ancestral exposure to aridity influences offspring water balance. Finally, we identify a critical question remaining unanswered: do hydroregulation strategies have an evolutionary limit? Studies on thermal tolerance suggest that plasticity alone may not be enough to ensure survival under extreme climate shifts (Morgan *et al.*, 2020), but we lack similar insights for hydroregulation. Addressing this could inform conservation strategies, helping predict whether species can adjust to future drying events or if their physiological flexibility has constraints.

### Modelling plasticity and adaptation in response to environmental drying

Animals can remodel their phenotype (physiology, morphology and behaviour) to maintain optimal performance across a broad range of environments. This plastic response is a well-recognized phenomenon in predicting adaptive responses to climate change (Seebacher *et al.*, 2015; Urban *et al.*, 2016). Models that incorporate plasticity or adaptation tend to better predict a species’ extinction risk or range contraction (Riddell *et al.*, 2018b; Kellermann *et al.*, 2020). Therefore, we encourage modelling studies to explicitly incorporate plasticity to provide realistic assessments of vulnerability to climate change (Bush *et al.*, 2016; Gallegos *et al.*, 2024). There is a substantial body of literature on plastic responses to pond drying in tadpoles (Gomez-Mestre *et al.*, 2013; Székely *et al.*, 2017; Delgadillo Méndez *et al.*, 2024; Wu *et al.*, 2025) and responses to soil moisture during embryo development in reptiles (reviewed in Bell *et al.*, 2025). However, in studies of terrestrial drying, many acclimation experiments aimed at quantifying plastic responses have primarily focused on temperature effects on hydroregulation traits. This can confound causal inferences between the effects of temperature and drying (see ‘Species sensitivity risk: long-term impacts’ section). For instance, the temperature effects on EWL may partially arise from the temperature-dependent nature of the metabolic rate (MR), as MR and the rate of gas exchange are closely linked to REWL (Woods and Smith, 2010), but see Riddell *et al.* (2024). It is possible that plasticity to water restrictions may differ from plasticity to temperature changes, potentially altering model predictions of extinction risk. Further studies across a broader range of species are needed to make biologically meaningful statements about the generality of within- and across-generation plasticity to drying, and to improve inferences in modelling vulnerability to future environmental drying scenarios. Finally, models explicitly testing adaptive evolution of traits should be more widely used (Hansen, 2012; Moen *et al.*, 2022).

### Translating knowledge for managing habitats

Incorporating knowledge on hydroregulation strategies with projected changes in environmental water into land management and conservation planning, particularly at the microhabitat scale, could help mitigate the impacts of habitat modification and climate change on reptiles and amphibians. Complex microhabitats, such as heterogeneous vegetation patches and burrows, provide hydric refuges during droughts which reptiles and amphibians can exploit through behavioural hydroregulation to avoid desiccation (see ‘Behavioural responses’ section). Indeed, incorporating species behaviour, such as moisture harvesting in tree frogs, for planning land management is important because this behaviour requires the presence of hollow trees (Tracy *et al.*, 2011). Conservation efforts should thus prioritize the protection, restoration or creation of such microhabitats within the range of the focal species to support persistence under increasingly drying conditions (Moore *et al.*, 2018; Weaver *et al.*, 2024). For example, protecting swamps from groundwater loss and surface water contamination has also been recommended for conserving *Eulamprus leuraensis*, an endangered swamp-specialist skink in Australia (Gorissen *et al.*, 2017). Interventions aimed at extending hydroperiods may protect some amphibians from larval desiccation and enhance population viability by increasing recruitment (Hamer *et al.*, 2016; Mathwin *et al.*, 2021; Mathwin *et al.*, 2023). Likewise, habitat water supplementation through mist irrigation can extend activity time in reptiles and amphibians under arid conditions (Ackley *et al.*, 2015; Mathwin *et al.*, 2021) and support reproduction and dispersal in amphibians (Mitchell, 2001; Channing *et al.*, 2006; Hoffmann and Mitchell, 2022). Providing supplemental hydration, such as drinking water for targeted individuals, could also be an effective conservation strategy for small, range-limited species (Weaver *et al.*, 2024), particularly benefiting gravid or pregnant females by mitigating physiological and reproductive costs during severe droughts (Capehart *et al.*, 2016; Dezetter *et al.*, 2021; Bedard *et al.*, 2025). However, the feasibility of such interventions is questionable (Mathwin *et al.*, 2021; Weaver *et al.*, 2024) and further research is needed to assess the effectiveness, potential negative and species-specific outcomes of hydrological manipulation and micro-habitat-scale management as a conservation tool. For example, modelling approaches can inform the optimal time for habitat water supplementation for the endangered southern bell frog *Litoria raniformis* while minimizing the risk of *Bd* infection (Turner *et al.*, 2025). To facilitate the global implementation of conservation, informed by recent and emerging hydroregulation research, publications are also needed in more accessible and taxa- and region-specific journals and government reports (Choi *et al.*, 2024; Amano and Berdejo-Espinola, 2025).

## Data Availability

No data were collected nor code developed for this review.

## References

[ref1] Abatzoglou JT, Dobrowski SZ, Parks SA, Hegewisch KC (2018) TerraClimate, a high-resolution global dataset of monthly climate and climatic water balance from 1958–2015. Sci Data 5: 170191. 10.1038/sdata.2017.191. 29313841 PMC5759372

[ref2] Ackley JW, Angilletta MJ, DeNardo D, Sullivan B, Wu J (2015) Urban heat island mitigation strategies and lizard thermal ecology: landscaping can quadruple potential activity time in an arid city. Urban Ecosyst 18: 1447–1459. 10.1007/s11252-015-0460-x.

[ref3] Adolph EF (1932) The vapor tension relations of frogs. Biol Bull 62: 112–125. 10.2307/1537147.

[ref4] Albecker MA, Strobel SM, Womack MC (2023) Developmental plasticity in anurans: meta-analysis reveals effects of larval environments on size at metamorphosis and timing of metamorphosis. Integr. Comp. Biol. 63: 714–729. 10.1093/icb/icad059. 37279893

[ref5] Amano T, Berdejo-Espinola V (2025) Language barriers in conservation: consequences and solutions. Trends Ecol Evol 40: 273–285. 10.1016/j.tree.2024.11.003. 39706729

[ref6] de Amaral M, Von Dentz MC, Cubas GK, de Oliveira DR, Simões LAR, Model JFA, Oliveira GT, Kucharski LC (2024) Coping with dry spells: investigating oxidative balance and metabolic responses in the subtropical tree frog *Boana pulchella* (Hylidae) during dehydration and rehydration exposure. Comp Biochem Physiol Part A Mol Integr Physiol 297: 111728. 10.1016/j.cbpa.2024.111728. 39147093

[ref7] Anderson DB (1936) Relative humidity or vapor pressure deficit. Ecology 17: 277–282. 10.2307/1931468.

[ref8] Anderson NL, Hetherington TE, Williams JB (2003) Validation of the doubly labeled water method under low and high humidity to estimate metabolic rate and water flux in a tropical snake (*Boiga irregularis*). J Appl Physiol 95: 184–191. 10.1152/japplphysiol.00692.2002. 12665536

[ref9] Anderson RC, Andrade DV (2017) Trading heat and hops for water: dehydration effects on locomotor performance, thermal limits, and thermoregulatory behavior of a terrestrial toad. Ecol Evol 7: 9066–9075. 10.1002/ece3.3219. 29152198 PMC5677477

[ref10] Anderson RC, Bovo RP, Eismann CE, Menegario AA, Andrade DV (2017) Not good, but not all bad: dehydration effects on body fluids, organ masses, and water flux through the skin of *Rhinella schneideri* (Amphibia, Bufonidae). Physiol Biochem Zool 90: 313–320. 10.1086/690189. 28384420

[ref11] Anderson RO, Tingley R, Hoskin CJ, White CR, Chapple DG (2023) Linking physiology and climate to infer species distributions in Australian skinks. J Anim Ecol 92: 2094–2108. 10.1111/1365-2656.14000. 37661659

[ref12] Araya-Donoso R, San Juan E, Tamburrino Í, Lamborot M, Veloso C, Véliz D (2022) Integrating genetics, physiology and morphology to study desert adaptation in a lizard species. J Anim Ecol 91: 1148–1162. 10.1111/1365-2656.13546. 34048024

[ref13] Beaman JE, White CR, Seebacher F (2016) Evolution of plasticity: mechanistic link between development and reversible acclimation. Trends Ecol Evol 31: 237–249. 10.1016/j.tree.2016.01.004. 26846962

[ref14] Beaupre SJ (1996) Field metabolic rate, water flux, and energy budgets of mottled rock rattlesnakes, *Crotalus lepidus*, from two populations. Copeia 1996: 319–329. 10.2307/1446847.

[ref15] Beck HE, Zimmermann NE, McVicar TR, Vergopolan N, Berg A, Wood EF (2018) Present and future Köppen-Geiger climate classification maps at 1-km resolution. Sci. Data 5: 1–12. 10.1038/sdata.2018.214. 30375988 PMC6207062

[ref16] Bedard RE, Weaver SJ, Moniz HA, Boback SM, Taylor EN (2025) Flexibility of cutaneous evaporative water loss in response to hydration in pregnant prairie rattlesnakes (*Crotalus viridis*) and their neonates. J Exp Biol 228: JEB247964. 10.1242/jeb.247964. 39670524

[ref17] Beissinger SR, Riddell EA (2021) Why are species’ traits weak predictors of range shifts? Annu Rev Ecol Evol S 52: 47–66. 10.1146/annurev-ecolsys-012021-092849.

[ref18] Bell C, Raynal RS, Noble DW, Schwanz LE, Warner DA, Pruett JE, Riley JL (2025) The effect of moisture during development on phenotypes of egg-laying reptiles: a systematic review and meta-analysis. J Exp Biol 228: JEB249960. 10.1242/jeb.249960. 39775816

[ref19] Benard MF (2004) Predator-induced phenotypic plasticity in organisms with complex life histories. Annu. Rev. Ecol. Evol. S. 35: 651–673. 10.1146/annurev.ecolsys.35.021004.112426.

[ref20] Bentley P, Main A (1972) Zonal differences in permeability of the skin of some anuran amphibia. Am J Physiol 223: 361–363. 10.1152/ajplegacy.1972.223.2.361. 4625782

[ref21] Bentley PJ, Schmidt-Nielsen K (1966) Cutaneous water loss in reptiles. Science 151: 1547–1549. 10.1126/science.151.3717.1547. 5909589

[ref22] Biber MF, Voskamp A, Hof C (2023) Potential effects of future climate change on global reptile distributions and diversity. Glob Ecol Biogeogr 32: 519–534. 10.1111/geb.13646.

[ref23] Birkmann, J. and McMillan, J. M., Birkmann J., McMillan J. M. (2020). Linking hazard vulnerability, risk reduction, and adaptation. In Oxford Research Encyclopedia of Natural Hazard Science eds. B.J. Gerber. Oxford University Press, Oxford, UK. 10.1093/acrefore/9780199389407.013.145.

[ref24] Bleu J, Massot M, Haussy C, Meylan S (2013) An experimental study of the gestation costs in a viviparous lizard: a hormonal manipulation. Physiol Biochem Zool 86: 690–701. 10.1086/673099. 24241066

[ref25] Bodensteiner BL, Gangloff EJ, Kouyoumdjian L, Muñoz MM, Aubret F (2021) Thermal–metabolic phenotypes of the lizard *Podarcis muralis* differ across elevation, but converge in high-elevation hypoxia. J Exp Biol 224: jeb243660. 10.1242/jeb.243660. 34761802

[ref26] Booth DT (2002) The double-labeled water technique is impractical for measurement of field metabolic rate in freshwater turtles. Herpetol Rev 33: 105.

[ref27] Bovo RP, Navas CA, Tejedo M, Valença SE, Gouveia SF (2018) Ecophysiology of amphibians: information for best mechanistic models. Diversity 10: 118. 10.3390/d10040118.

[ref28] Bovo RP, Simon MN, Provete DB, Lyra M, Navas CA, Andrade DV (2023) Beyond Janzen's hypothesis: how amphibians that climb tropical mountains respond to climate variation. Integr Org Biol 5: obad009. 10.1093/iob/obad009. 37151602 PMC10155226

[ref29] Bradshaw SD (2012) Homeostasis in Desert Reptiles. Springer Berlin, Heidelberg.

[ref30] Bramer, I., Anderson, B. J., Bennie, J., Bladon, A. J., De Frenne, P., Hemming, D., Hill, R. A., Kearney, M. R., Körner, C. and Korstjens, A. H. (2018). Advances in monitoring and modelling climate at ecologically relevant scales. In Adv. Ecol. Res*.*, vol. 58 eds. D. A. Bohan, A. J. Dumbrell, G. Woodward and M. Jackson, pp. 101–161. Elsevier, Amsterdam, Netherlands.

[ref31] Brannelly LA, Martin G, Llewelyn J, Skerratt LF, Berger L (2018) Age-and size-dependent resistance to chytridiomycosis in the invasive cane toad *Rhinella marina*. Dis Aquat Organ 131: 107–120. 10.3354/dao03278. 30460917

[ref32] Brannelly LA, Ohmer ME, Saenz V, Richards-Zawacki CL (2019) Effects of hydroperiod on growth, development, survival, and immune defenses in a temperate amphibian. Funct. Ecol. 33: 1952–1961. 10.1111/1365-2435.13419.

[ref33] Brischoux F, Cheron M (2019) Osmotic ‘cost’of reproduction in breeding male toads. Biol Lett 15: 20190689. 10.1098/rsbl.2019.0689. 31718512 PMC6892512

[ref34] Brischoux F, Gartner GE, Garland T Jr, Bonnet X (2011) Is aquatic life correlated with an increased hematocrit in snakes? PloS One 6: e17077. 10.1371/journal.pone.0017077. 21359216 PMC3040194

[ref35] Brischoux F, Kornilev YV, Lillywhite HB (2017) Physiological and behavioral responses to salinity in coastal dice snakes. Comp. Biochem. Physiol. Part A Mol. Integr. Physiol. 214: 13–18. 10.1016/j.cbpa.2017.09.003. 28893666

[ref36] Briscoe NJ, Morris SD, Mathewson PD, Buckley LB, Jusup M, Levy O, Maclean IM, Pincebourde S, Riddell EA, Roberts JA et al. (2023) Mechanistic forecasts of species responses to climate change: the promise of biophysical ecology. Glob Chang Biol 29: 1451–1470. 10.1111/gcb.16557. 36515542

[ref37] Brun P, Zimmermann NE, Hari C, Pellissier L, Karger DN (2022) Global climate-related predictors at kilometer resolution for the past and future. Earth Syst Sci Data 14: 5573–5603. 10.5194/essd-14-5573-2022.

[ref38] Brusch GA, DeNardo DF (2017) When less means more: dehydration improves innate immunity in rattlesnakes. J Exp Biol 220: 2287–2295. 10.1242/jeb.155028. 28404727

[ref39] Brusch GA, DeNardo DF, Lourdais O (2020) Reproductive state and water deprivation increase plasma corticosterone in a capital breeder. Gen Comp Endocrinol 288: 113375. 10.1016/j.ygcen.2019.113375. 31874136

[ref40] Brusch GA, Heulin B, DeNardo DF (2019) Dehydration during egg production alters egg composition and yolk immune function. Comp. Biochem. Physiol. Part A Mol. Integr. Physiol. 227: 68–74. 10.1016/j.cbpa.2018.10.006. 30300746

[ref41] Brusch GA, Lourdais O, Kaminsky B, DeNardo DF (2018) Muscles provide an internal water reserve for reproduction. Proc R Soc B 285: 20180752. 10.1098/rspb.2018.0752. PMC6030541 30051850

[ref42] Buchmiller NE, Weaver SJ, Bedard RE, Taylor EN, Moniz HA (2024) Storage time and temperature affect plasma osmolality values in field-collected blood samples. Comp. Biochem. Physiol. Part A Mol. Integr. Physiol. 295: 111665. 10.1016/j.cbpa.2024.111665. 38762048

[ref43] Bulova SJ (2002) How temperature, humidity, and burrow selection affect evaporative water loss in desert tortoises. J Therm Biol 27: 175–189. 10.1016/S0306-4565(01)00079-1.

[ref44] Bush A, Mokany K, Catullo R, Hoffmann A, Kellermann V, Sgrò C, McEvey S, Ferrier S (2016) Incorporating evolutionary adaptation in species distribution modelling reduces projected vulnerability to climate change. Ecol Lett 19: 1468–1478. 10.1111/ele.12696. 27873482

[ref45] Bustin SA, Benes V, Garson JA, Hellemans J, Huggett J, Kubista M, Mueller R, Nolan T, Pfaffl MW, Shipley GL et al. (2009) The MIQE guidelines: minimum information for publication of quantitative real-time PCR experiments. Clin Chem 55: 611–622. 10.1373/clinchem.2008.112797. 19246619

[ref46] Cabrera-Guzmán E, Crossland MR, Brown GP, Shine R (2013) Larger body size at metamorphosis enhances survival, growth and performance of young cane toads (*Rhinella marina*). PloS One 8: e70121. 10.1371/journal.pone.0070121. 23922930 PMC3726449

[ref47] Caine ST, Mclaughlin KA (2013) Regeneration of functional pronephric proximal tubules after partial nephrectomy in *Xenopus laevis*. Dev Dyn 242: 219–229. 10.1002/dvdy.23916. 23233460

[ref48] Calosi P, Bilton DT, Spicer JI (2008) Thermal tolerance, acclimatory capacity and vulnerability to global climate change. Biol Lett 4: 99–102. 10.1098/rsbl.2007.0408. 17986429 PMC2412917

[ref49] Camacho A, Brunes TO, Rodrigues MT (2023) Dehydration alters behavioral thermoregulation and the geography of climatic vulnerability in two Amazonian lizards. PloS One 18: e0286502. 10.1371/journal.pone.0286502. 37910524 PMC10619801

[ref50] Cameron L, Mills B, Peters A (2024) Plasma osmolality of Australian reptiles: are we assuming too much? J. Herpetol. Med. Surg. 34: 193–198. 10.5818/JHMS-D-23-00026.

[ref51] Campbell GS, Norman JM (2000) An introduction to environmental biophysics. Springer, Berlin, Germany.

[ref52] Capehart GD, Escallón C, Vernasco BJ, Moore IT, Taylor EN (2016) No drought about it: effects of supplemental hydration on the ecology, behavior, and physiology of free-ranging rattlesnakes. J Arid Environ 134: 79–86. 10.1016/j.jaridenv.2016.06.018.

[ref53] Carsia RV, McIlroy PJ, John-Alder HB (2023) Invited review: adrenocortical function in avian and non-avian reptiles: insights from dispersed adrenocortical cells. Comp. Biochem. Physiol. Part A Mol. Integr. Physiol. 281: 111424. 10.1016/j.cbpa.2023.111424. 37080352

[ref54] Carvalho, J. E., Navas, C. A. and Pereira, I. C. (2010). Energy and water in aestivating amphibians. In Aestivation: Molecular and Physiological Aspects, eds. C. A. Navas and J. E. Carvalho), pp. 141–169. Heidelberg, Germany: Springer, 10.1007/978-3-642-02421-4_7. 20069408

[ref55] Chabaud C, Brusch GA, Pellerin A, Lourdais O, Le Galliard JF (2023) Prey consumption does not restore hydration state but mitigates the energetic costs of water deprivation in an insectivorous lizard. J Exp Biol 226: jeb246129. 10.1242/jeb.246129. 37577990

[ref56] Chahine MT (1992) The hydrological cycle and its influence on climate. Nature 359: 373–380. 10.1038/359373a0.

[ref57] Channing A, Finlow-Bates KS, Haarklau SE, Hawkes PG (2006) The biology and recent history of the critically endangered Kihansi spray toad *Nectophrynoides asperginis* in Tanzania. J East Afr Nat Hist 95: 117–138. 10.2982/0012-8317(2006)95[117:TBARHO]2.0.CO;2.

[ref58] Charbonnier JF, Pearlmutter J, Vonesh JR, Gabor CR, Forsburg ZR, Grayson KL (2018) Cross-life stage effects of aquatic larval density and terrestrial moisture on growth and corticosterone in the spotted salamander. Diversity 10: 68. 10.3390/d10030068.

[ref59] Chen J, Zhong H, Ren J, Zhao W, Man Q, Shang S, Tang X (2019) Genome-wide analysis of the Aquaporin gene family in reptiles. Int J Biol Macromol 126: 1093–1098. 10.1016/j.ijbiomac.2019.01.007. 30611807

[ref60] Cheng CT, Chuang MF, Haramura T, Cheng CB, Kim YI, Borzée A, Wu CS, Chen YH, Jang Y, Wu NC et al. (2023) Open habitats increase vulnerability of amphibian tadpoles to climate warming across latitude. Glob Ecol Biogeogr 32: 83–94. 10.1111/geb.13602.

[ref61] Choi JJ, Gaskins LC, Morton JP, Bingham JA, Blawas AM, Hayes C, Hoyt C, Halpin PN, Silliman B (2024) Role of low-impact-factor journals in conservation implementation. Conserv Biol 39: e14391. 39417626 10.1111/cobi.14391 PMC11959337

[ref62] Chown SL, Sørensen JG, Terblanche JS (2011) Water loss in insects: an environmental change perspective. J Insect Physiol 57: 1070–1084. 10.1016/j.jinsphys.2011.05.004. 21640726

[ref63] Christian K, Bedford G, Green B, Griffiths A, Newgrain K, Schultz T (1999) Physiological ecology of a tropical dragon, *Lophognathus temporalis*. Aust J Ecol 24: 171–181. 10.1046/j.1442-9993.1999.241960.x.

[ref64] Christian K, Green B (1994) Water flux in the Australian treefrog *Litoria caerulea* under natural and semi-natural conditions. Amphib-Reptilia 15: 401–405. 10.1163/156853894X00434.

[ref65] Christian K, Webb JK, Schultz T, Green B (2007) Effects of seasonal variation in prey abundance on field metabolism, water flux, and activity of a tropical ambush foraging snake. Physiol Biochem Zool 80: 522–533. 10.1086/519959. 17717815

[ref66] Christian KA, Bedford G, Green B, Schultz T, Newgrain K (1998) Energetics and water flux of the marbled velvet gecko (*Oedura marmorata*) in tropical and temperate habitats. Oecologia 116: 336–342. 10.1007/s004420050595. 28308064

[ref67] Cloudsley-Thompson, J. L., Cloudsley-Thompson J. L. (1999). Water balance and excretion. In The Diversity of Amphibians and Reptiles: An Introduction, (ed. J. L. Cloudsley-Thompson), pp. 197–212. Heidelberg, Germany: Springer Berlin, 10.1007/978-3-642-60005-0_11.

[ref68] Coelho MTP, Barreto E, Rangel TF, Diniz-Filho JAF, Wüest RO, Bach W, Skeels A, McFadden IR, Roberts DW, Pellissier L et al. (2023) The geography of climate and the global patterns of species diversity. Nature 622: 537–544. 10.1038/s41586-023-06577-5. 37758942 PMC10584679

[ref69] Cohen NW (1952) Comparative rates of dehydration and hydration in some California salamanders. Ecology 33: 462–479. 10.2307/1931521.

[ref70] Comanns P, Esser FJ, Kappel PH, Baumgartner W, Shaw J, Withers PC (2017) Adsorption and movement of water by skin of the Australian thorny devil (Agamidae: *Moloch horridus*). Roy Soc Open Sci 4: 170591. 10.1098/rsos.170591. 28989762 PMC5627102

[ref71] Cooke SJ, Sack L, Franklin CE, Farrell AP, Beardall J, Wikelski M, Chown SL (2013) What is conservation physiology? Perspectives on an increasingly integrated and essential science. Conserv. Physiol. 1: cot001. 10.1093/conphys/cot001. 27293585 PMC4732437

[ref72] Cox CL, Cox RM (2015) Evolutionary shifts in habitat aridity predict evaporative water loss across squamate reptiles. Evolution 69: 2507–2516. 10.1111/evo.12742. 26227547

[ref73] Crausbay SD, Hall KR, Cross MS, Halabisky M, Rangwala I, Anderson J, Schwend A (2024) A flexible data-driven approach to co-producing drought vulnerability assessments. Ecosphere 15: e70040. 10.1002/ecs2.70040.

[ref74] Crespi EJ, Warne RW (2013) Environmental conditions experienced during the tadpole stage alter post-metamorphic glucocorticoid response to stress in an amphibian. Integr. Comp. Biol. 53: 989–1001. 10.1093/icb/ict087. 23922274

[ref75] Crino OL, Wild KH, Friesen CR, Leibold D, Laven N, Peardon AY, Recio P, Salin K, Noble DW (2024) From eggs to adulthood: sustained effects of early developmental temperature and corticosterone exposure on physiology and body size in an Australian lizard. J Exp Biol 227: jeb249234. 10.1242/jeb.249234. 39665281 PMC11655029

[ref76] Cruz-Piedrahita C, Navas CA, Crawford AJ (2018) Life on the edge: a comparative study of ecophysiological adaptations of frogs to tropical semiarid environments. Physiol Biochem Zool 91: 740–756. 10.1086/695705. 29211619

[ref77] Dai A, Trenberth KE, Qian T (2004) A global dataset of Palmer Drought Severity Index for 1870–2002: relationship with soil moisture and effects of surface warming. J Hydrometeorol 5: 1117–1130. 10.1175/JHM-386.1.

[ref78] D'Alba L, Goldenberg J, Nallapaneni A, Parkinson DY, Zhu C, Vanthournout B, Shawkey MD (2021) Evolution of eggshell structure in relation to nesting ecology in non-avian reptiles. J Morphol 282: 1066–1079. 10.1002/jmor.21347. 33713039

[ref79] Dallwig RK, Mitchell MA, Acierno MJ (2010) Determination of plasma osmolality and agreement between measured and calculated values in healthy adult bearded dragons (*Pogona vitticeps*). J Herpetol Med Surg 20: 69–73. 10.5818/1529-9651-20.2.69.

[ref80] Daltry JC, Ross T, Thorpe RS, Wüster W (1998) Evidence that humidity influences snake activity patterns: a field study of the Malayan pit viper *Calloselasma rhodostoma*. Ecography 21: 25–34. 10.1111/j.1600-0587.1998.tb00391.x.

[ref81] Dantzler WH, Bradshaw SD (2008) Osmotic and ionic regulation in reptiles. In DH Evans, ed, Osmotic and Ionic Regulation: Cells and Animals. CRC Press, Boca Raton, pp. 443–503

[ref82] Davies SJ, McGeoch MA, Clusella-Trullas S (2015) Plasticity of thermal tolerance and metabolism but not water loss in an invasive reed frog. Comp. Biochem. Physiol. Part A Mol. Integr. Physiol. 189: 11–20. 10.1016/j.cbpa.2015.06.033. 26164532

[ref83] Davis JR, DeNardo DF (2010) Seasonal patterns of body condition, hydration state, and activity of Gila monsters (*Heloderma suspectum*) at a Sonoran Desert site. J Herpetol 44: 83–93. 10.1670/08-263.1.

[ref84] Day K, Weitzman CL, Rachmansah A, Skelton K, Christian K (2025) Patterns of seasonal plasticity in evaporative water loss and preferred temperature in three geckos of the wet–dry tropics. Oecologia 207: 53. 10.1007/s00442-025-05692-6. 40085226 PMC11909027

[ref85] De Frenne P, Beugnon R, Klinges D, Lenoir J, Niittynen P, Pincebourde S, Senior RA, Aalto J, Chytrý K, Gillingham PK (2025) Ten practical guidelines for microclimate research in terrestrial ecosystems. Method Ecol Evol 16: 269–294.

[ref86] Debruyn G, Geltmeyer J, Schoolaert E, Nicolaï MP, Xie W, Wynant M, Shawkey MD, De Clerck K, D'Alba L (2023) Hydric environment and chemical composition shape non-avian reptile eggshell absorption. Integr Comp Biol 64: 107–119. 10.1093/icb/icae040 38755009

[ref87] Delgadillo Méndez A, Amézquita A, Avellaneda Moreno MA, González-Arango C, Gomez-Mestre I (2024) Developmental plasticity to desiccation risk in tadpoles of a tropical inselberg specialist. Front Ecol Evol 12: 1370932. 10.3389/fevo.2024.1370932.

[ref88] Denver RJ (1997) Environmental stress as a developmental cue: corticotropin-releasing hormone is a proximate mediator of adaptive phenotypic plasticity in amphibian metamorphosis. Horm Behav 31: 169–179. 10.1006/hbeh.1997.1383. 9154437

[ref89] Denver RJ (2013) Neuroendocrinology of amphibian metamorphosis. Curr Top Dev Biol 103: 195–227. 10.1016/B978-0-12-385979-2.00007-1. 23347520

[ref90] Dessauer HC (1970) Blood chemistry of reptiles: physiological and evolutionary aspects. In C Gans, ed, Biology of the Reptilia. Volume 3. Morphology C. Academic Press, London, pp. 1–72.

[ref91] Deutsch CA, Tewksbury JJ, Huey RB, Sheldon KS, Ghalambor CK, Haak DC, Martin PR (2008) Impacts of climate warming on terrestrial ectotherms across latitude. Proc Natl Acad Sci 105: 6668–6672. 10.1073/pnas.0709472105. 18458348 PMC2373333

[ref92] Dezetter M, Dupoué A, Le Galliard JF, Lourdais O (2022a) Additive effects of developmental acclimation and physiological syndromes on lifetime metabolic and water loss rates of a dry-skinned ectotherm. Funct Ecol 36: 432–445. 10.1111/1365-2435.13951.

[ref93] Dezetter M, Le Galliard JF, Guiller G, Guillon M, Leroux-Coyau M, Meylan S, Brischoux F, Angelier F, Lourdais O (2021) Water deprivation compromises maternal physiology and reproductive success in a cold and wet adapted snake *Vipera berus*. Conserv. Physiol. 9: coab071. 10.1093/conphys/coab071. 34512993 PMC8415537

[ref94] Dezetter M, Le Galliard JF, Leroux-Coyau M, Brischoux F, Angelier F, Lourdais O (2022b) Two stressors are worse than one: combined heatwave and drought affect hydration state and glucocorticoid levels in a temperate ectotherm. J Exp Biol 225: jeb243777. 10.1242/jeb.243777. 35319758

[ref95] Dezetter M, Le Galliard JF, Lourdais O (2023) Behavioural hydroregulation protects against acute effects of drought in a dry-skinned ectotherm. Oecologia 201: 355–367. 10.1007/s00442-022-05299-1. 36564481

[ref96] Dial, K. P., Shubin, N. and Brainerd, E. L. (2015). Great Transformations in Vertebrate Evolution. Chicago: University of Chicago Press, 10.7208/chicago/9780226268392.001.0001.

[ref97] Dmiel R (2001) Skin resistance to evaporative water loss in reptiles: a physiological adaptive mechanism to environmental stress or a phyletically dictated trait? Isr J Zool 47: 56–67. 10.1092/ENQ9-KD7R-WFGW-KUQW.

[ref98] Dubiner S, Jamison S, Meiri S, Levin E (2023) Squamate metabolic rates decrease in winter beyond the effect of temperature. J Anim Ecol 92: 2163–2174. 10.1111/1365-2656.13997. 37632258

[ref99] Dupoué A, Angelier F, Ribout C, Meylan S, Rozen-Rechels D, Decencière B, Agostini S, Le Galliard JF (2020b) Chronic water restriction triggers sex-specific oxidative stress and telomere shortening in lizards. Biol Lett 16: 20190889. 10.1098/rsbl.2019.0889. 32097601 PMC7058957

[ref100] Dupoué A, Blaimont P, Rozen-Rechels D, Richard M, Meylan S, Clobert J, Miles DB, Martin R, Decencière B, Agostini S et al. (2020c) Water availability and temperature induce changes in oxidative status during pregnancy in a viviparous lizard. Funct. Ecol. 34: 475–485. 10.1111/1365-2435.13481.

[ref101] Dupoue A, Brischoux F, Angelier F, DeNardo DF, Wright CD, Lourdais O (2015) Intergenerational trade-off for water may induce a mother–offspring conflict in favour of embryos in a viviparous snake. Funct. Ecol. 29: 414–422. 10.1111/1365-2435.12349.

[ref102] Dupoué A, Le Galliard JF, Josserand R, DeNardo DF, Decencière B, Agostini S, Haussy C, Meylan S (2018) Water restriction causes an intergenerational trade-off and delayed mother–offspring conflict in a viviparous lizard. Funct. Ecol. 32: 676–686. 10.1111/1365-2435.13009.

[ref103] Dupoué A, Rutschmann A, Le Galliard JF, Miles DB, Clobert J, DeNardo DF, Brusch GA, Meylan S (2017) Water availability and environmental temperature correlate with geographic variation in water balance in common lizards. Oecologia 185: 561–571. 10.1007/s00442-017-3973-6. 29018996

[ref104] Dupoué A, Sorlin M, Richard M, Le Galliard JF, Lourdais O, Clobert J, Aubret F (2020a) Mother-offspring conflict for water and its mitigation in the oviparous form of the reproductively bimodal lizard, *Zootoca vivipara*. Biol J Linn Soc 129: 888–900. 10.1093/biolinnean/blaa012.

[ref105] Dupoué A, Stahlschmidt ZR, Michaud B, Lourdais O (2015) Physiological state influences evaporative water loss and microclimate preference in the snake *Vipera aspis*. Physiol Behav 144: 82–89. 10.1016/j.physbeh.2015.02.042. 25725119

[ref106] Dupoué AA, Angelier FDR, Brischoux F, DeNardo DF, Trouvé C, Parenteau C, Lourdais O (2016) Water deprivation increases maternal corticosterone levels and enhances offspring growth in the snake *Vipera aspis*. J Exp Biol 219: 658–667. 10.1242/jeb.132639. 26747902

[ref107] Edwards M, Sheehy CM 3rd, Fedler MT, Lillywhite HB (2021) Thirst and drinking in North American watersnakes (*Nerodia* spp.). J Exp Biol 224: jeb241414. 10.1242/jeb.241414. 33674397 PMC7938798

[ref108] Elith J, Leathwick JR (2009) Species distribution models: ecological explanation and prediction across space and time. Annu Rev Ecol Evol S 40: 677–697. 10.1146/annurev.ecolsys.110308.120159.

[ref109] Encarnación-Luévano A, Peterson AT, Rojas-Soto OR (2021) Burrowing habit in Smilisca frogs as an adaptive response to ecological niche constraints in seasonally dry environments. Frontiers of Biogeography 13: e50517. 10.21425/F5FBG50517.

[ref110] Enriquez-Urzelai U, Kearney MR, Nicieza AG, Tingley R (2019) Integrating mechanistic and correlative niche models to unravel range-limiting processes in a temperate amphibian. Glob Chang Biol 25: 2633–2647. 10.1111/gcb.14673. 31050846

[ref111] Fanter C, Madelaire C, Genereux DP, van Breukelen F, Levesque D, Hindle A (2022) Epigenomics as a paradigm to understand the nuances of phenotypes. J Exp Biol 225: jeb243411. 10.1242/jeb.243411. 35258621

[ref112] Feder ME, Burggren WW (1992) Environmental Physiology of the Amphibians. University of Chicago Press, Chicago, USA

[ref113] Fick SE, Hijmans RJ (2017) WorldClim 2: new 1-km spatial resolution climate surfaces for global land areas. Int J Clim 37: 4302–4315. 10.1002/joc.5086.

[ref114] Foley RE, Spotila JR (1978) Effect of wind speed, air temperature, body size and vapor density difference on evaporative water loss from the turtle *Chrysemys scripta*. Copeia 1978: 627–634. 10.2307/1443689.

[ref115] Funk C, Peterson P, Landsfeld M, Pedreros D, Verdin J, Shukla S, Husak G, Rowland J, Harrison L, Hoell A et al. (2015) The climate hazards infrared precipitation with stations—a new environmental record for monitoring extremes. Sci Data 2: 1–21. 10.1038/sdata.2015.66. PMC4672685 26646728

[ref116] Galindo C, Gutiérrez K, Calvache L, Bernal M (2024) Effect of hydration state on locomotor performance and water searching behavior of the terrestrial lungless salamander *Bolitoglossa ramosi*. J Zool 322: 35–41. 10.1111/jzo.13121.

[ref117] Gallegos C, Chevin LM, Hodgins KA, Monro K (2024) Predicting adaptation and evolution of plasticity from temporal environmental change. Method. Ecol. Evol. 16: 84–96. 10.1111/2041-210X.14462.

[ref118] Galloway LF, Etterson JR (2007) Transgenerational plasticity is adaptive in the wild. Science 318: 1134–1136. 10.1126/science.1148766. 18006745

[ref119] Gates, D. M. (1980). Biophysical Ecology. New York, USA: Springer-Verlag New York, 10.1007/978-1-4612-6024-0.

[ref120] Gatto CR, Reina RD (2022) A review of the effects of incubation conditions on hatchling phenotypes in non-squamate reptiles. J Comp Physiol B 192: 207–233. 10.1007/s00360-021-01415-4. 35142902 PMC8894305

[ref121] Geiger R, Aron RH, Todhunter P (2003) The Climate Near the Ground. Springer, Braunschweig, Germany

[ref122] Gervasi SS, Foufopoulos J (2008) Costs of plasticity: responses to desiccation decrease post-metamorphic immune function in a pond-breeding amphibian. Funct. Ecol. 22: 100–108. 10.1111/j.1365-2435.2007.01340.x.

[ref123] Giacometti D, Tattersall GJ (2023) Putting the energetic-savings hypothesis underground: fossoriality does not affect metabolic rates in amphibians. Evol Ecol 37: 761–777. 10.1007/s10682-023-10253-5.

[ref124] Glaudas X (2009) Rain-harvesting by the southwestern speckled rattlesnake (*Crotalus mitchellii pyrrhus*). The Southwestern Naturalist 54: 518–521. 10.1894/WL-23.1.

[ref125] Gomez-Mestre I, Kulkarni S, Buchholz DR (2013) Mechanisms and consequences of developmental acceleration in tadpoles responding to pond drying. PloS One 8: e84266. 10.1371/journal.pone.0084266. 24358352 PMC3865288

[ref126] Gorissen S, Greenlees M, Shine R (2017) A skink out of water: impacts of anthropogenic disturbance on an endangered reptile in Australian highland swamps. Oryx 51: 610–618. 10.1017/S0030605316000442.

[ref127] Gould J, Clulow J, Clulow S (2022) High clutch failure rate due to unpredictable rainfall for an ephemeral pool-breeding frog. Oecologia 198: 699–710. 10.1007/s00442-022-05139-2. 35247072 PMC8956532

[ref128] Gouveia SF, Correia I (2016) Geographical clines of body size in terrestrial amphibians: water conservation hypothesis revisited. J Biogeogr 43: 2075–2084.

[ref129] Gray J (1928) The role of water in the evolution of the terrestrial vertebrates. J Exp Biol 6: 26–31. 10.1242/jeb.6.1.26.

[ref130] Greenberg DA, Palen WJ (2021) Hydrothermal physiology and climate vulnerability in amphibians. Proc R Soc B 288: 20202273. 10.1098/rspb.2020.2273. PMC7934955 33593188

[ref131] Greene HW (2005) Organisms in nature as a central focus for biology. Trends Ecol Evol 20: 23–27. 10.1016/j.tree.2004.11.005. 16701336

[ref132] Grubb JC (1973) Olfactory orientation in *Bufo woodhousei fowleri*, *Pseudacris clarki* and *Pseudacris streckeri*. Anim Behav 21: 726–732. 10.1016/S0003-3472(73)80098-3. 4777202

[ref133] Guo C, Gao S, Krzton A, Zhang L (2019) Geographic body size variation of a tropical anuran: effects of water deficit and precipitation seasonality on Asian common toad from southern Asia. BMC Evol Biol 19: 1–11. 10.1186/s12862-019-1531-z. 31706264 PMC6842474

[ref134] Hamer AJ, Heard GW, Urlus J, Ricciardello J, Schmidt B, Quin D, Steele WK (2016) Manipulating wetland hydroperiod to improve occupancy rates by an endangered amphibian: modelling management scenarios. J Appl Ecol 53: 1842–1851. 10.1111/1365-2664.12729.

[ref135] Hansen TF (2012) Adaptive landscapes and macroevolutionary dynamics. In E Svensson, R Calsbeek, eds, The Adaptive Landscape in Evolutionary Biology. Oxford University Press, Oxford, UK, pp. 205–226

[ref136] Harden LA, Duernberger KA, Jones TT, Williard AS (2014) Total body water and water turnover rates in the estuarine diamondback terrapin (*Malaclemys terrapin*) during the transition from dormancy to activity. J Exp Biol 217: 4406–4413. 10.1242/jeb.110411. 25394625

[ref137] Herrando-Pérez S, Ferri-Yáñez F, Monasterio C, Beukema W, Gomes V, Belliure J, Chown SL, Vieites DR, Araújo MB (2019) Intraspecific variation in lizard heat tolerance alters estimates of climate impact. J Anim Ecol 88: 247–257. 10.1111/1365-2656.12914. 30303530

[ref138] Herrando-Pérez S, Vieites DR, Araújo MB (2023) Novel physiological data needed for progress in global change ecology. Basic Appl Ecol 67: 32–47. 10.1016/j.baae.2023.01.002.

[ref139] Higginson A, Ruxton G (2010) Adaptive changes in size and age at metamorphosis can qualitatively vary with predator type and available defenses. Ecology 91: 2756–2768. 10.1890/08-2269.1. 20957968

[ref140] Hillman SS, Withers PC, Drewes RC, Hillyard SD (2009) Ecological and environmental physiology of amphibians. Oxford University Press, New York, USA

[ref141] Hillyard SD, Hoff K, v. S. and Propper, C. (1998) The water absorption response: a behavioral assay for physiological processes in terrestrial amphibians. Physiol Biochem Zool 71: 127–138. 10.1086/515900. 9548645

[ref142] Hoffmann EP, Mitchell NJ (2022) Breeding phenology of a terrestrial-breeding frog is associated with soil water potential: implications for conservation in a changing climate. Austral Ecol 47: 353–364. 10.1111/aec.13122.

[ref143] Hogg CJ (2024) Translating genomic advances into biodiversity conservation. Nat Rev Genet 25: 362–373. 10.1038/s41576-023-00671-0. 38012268

[ref144] Holthaus KB, Eckhart L (2024) Development-associated genes of the epidermal differentiation complex (EDC). J Dev Biol 12: 4. 10.3390/jdb12010004. 38248869 PMC10801484

[ref145] Holthaus KB, Sachslehner AP, Steinbinder J, Eckhart L (2024) Epidermal differentiation genes of the common wall lizard encode proteins with extremely biased amino acid contents. Genes 15: 1136. 10.3390/genes15091136. 39336727 PMC11431283

[ref146] Husby A (2022) Wild epigenetics: insights from epigenetic studies on natural populations. Proc R Soc B 289: 20211633. 10.1098/rspb.2021.1633. PMC8826306 35135348

[ref147] Joel, A.-C., Buchberger, G. and Comanns, P. (2017). Moisture-harvesting reptiles: a review. In Functional Surfaces in Biology III: Diversity of the Physical Phenomena, eds. S. N. Gorb and E. V. Gorb), pp. 93–106: Springer, Berline, Germany. 10.1007/978-3-319-74144-4_4.

[ref148] Jones TT, Hastings MD, Bostrom BL, Andrews RD, Jones DR (2009) Validation of the use of doubly labeled water for estimating metabolic rate in the green turtle (*Chelonia mydas* L.): a word of caution. J Exp Biol 212: 2635–2644. 10.1242/jeb.029330. 19648409

[ref149] Karger DN, Wilson AM, Mahony C, Zimmermann NE, Jetz W (2021) Global daily 1 km land surface precipitation based on cloud cover-informed downscaling. Sci. Data 8: 307. 10.1038/s41597-021-01084-6. 34836980 PMC8626457

[ref150] Kattan GH, Lillywhite HB (1989) Humidity acclimation and skin permeability in the lizard *Anolis carolinensis*. Physiol Zool 62: 593–606. 10.1086/physzool.62.2.30156187.

[ref151] Kearney MR, Enriquez-Urzelai U (2023) A general framework for jointly modelling thermal and hydric constraints on developing eggs. Method Ecol Evol 14: 583–595. 10.1111/2041-210X.14018.

[ref152] Kearney MR, Munns SL, Moore D, Malishev M, Bull CM (2018) Field tests of a general ectotherm niche model show how water can limit lizard activity and distribution. Ecological monographs 88: 672–693. 10.1002/ecm.1326.

[ref153] Kearney MR, Porter W (2009) Mechanistic niche modelling: combining physiological and spatial data to predict species’ ranges. Ecol Lett 12: 334–350. 10.1111/j.1461-0248.2008.01277.x. 19292794

[ref154] Kearney MR, Porter WP (2017) NicheMapR–an R package for biophysical modelling: the microclimate model. Ecography 40: 664–674. 10.1111/ecog.02360.

[ref155] Kearney MR, Porter WP (2020) NicheMapR–an R package for biophysical modelling: the ectotherm and dynamic energy budget models. Ecography 43: 85–96. 10.1111/ecog.04680.

[ref156] Kearney MR, Simpson SJ, Raubenheimer D, Kooijman SA (2013) Balancing heat, water and nutrients under environmental change: a thermodynamic niche framework. Funct. Ecol. 27: 950–966. 10.1111/1365-2435.12020.

[ref157] Kellermann V, McEvey SF, Sgrò CM, Hoffmann AA (2020) Phenotypic plasticity for desiccation resistance, climate change, and future species distributions: will plasticity have much impact? Am Nat 196: 306–315. 10.1086/710006. 32814000

[ref158] Kemppinen J, Lembrechts JJ, Van Meerbeek K, Carnicer J, Chardon NI, Kardol P, Lenoir J, Liu D, Maclean I, Pergl J (2024) Microclimate, an important part of ecology and biogeography. Glob Ecol Biogeogr 33: e13834. 10.1111/geb.13834.

[ref159] Kennett R, Christian K, Pritchard D (1993) Underwater nesting by the tropical fresh-water turtle, *Chelodina rugosa* (Testudinata, Chelidae). Aust J Zool 41: 47–52. 10.1071/ZO9930047.

[ref160] Kikuyama S, Kawamura K, Tanaka S, Yamamoto K (1993) Aspects of amphibian metamorphosis: hormonal control. Int Rev Cytol 145: 105–148. 10.1016/S0074-7696(08)60426-X. 8500980

[ref161] Kinlaw A (1999) A review of burrowing by semi-fossorial vertebrates in arid environments. J Arid Environ 41: 127–145. 10.1006/jare.1998.0476.

[ref162] Kirschman LJ, McCue MD, Boyles JG, Warne RW (2017) Exogenous stress hormones alter energetic and nutrient costs of development and metamorphosis. J Exp Biol 220: 3391–3397. 10.1242/jeb.164830. 28729344

[ref163] Klinges DH, Baecher JA, Lembrechts JJ, Maclean IM, Lenoir J, Greiser C, Ashcroft M, Evans LJ, Kearney MR, Aalto J et al. (2024) Proximal microclimate: moving beyond spatiotemporal resolution improves ecological predictions. Glob Ecol Biogeogr 33: e13884. 10.1111/geb.13884.

[ref164] Kobayashi D, Mautz WJ, Nagy KA (1983) Evaporative water loss: humidity acclimation in *Anolis carolinensis* lizards. Copeia 1983: 701–704. 10.2307/1444335.

[ref165] Kurta A (2014) The misuse of relative humidity in ecological studies of hibernating bats. Acta Chiropt 16: 249–254. 10.3161/150811014X683444.

[ref166] Ladyman M, Bradshaw D (2003) The influence of dehydration on the thermal preferences of the Western tiger snake. Notechis scutatus J Comp Physiol B 173: 239–246. 10.1007/s00360-003-0328-x. 12743727

[ref167] Ladyman M, Bradshaw D, Bradshaw F (2006) Physiological and hormonal control of thermal depression in the tiger snake. Notechis scutatus J Comp Physiol B 176: 547–557. 10.1007/s00360-006-0077-8. 16520994

[ref168] Lai S-J, Kam Y-C, Hsu F-H, Lin Y-S (2002) Elevational effects on the growth and development of tadpoles of Sauter’s frog *Rana sauteri* Boulenger in Taiwan. Acta Zoologica Taiwanica 13: 11–20.

[ref169] Le Galliard JF, Chabaud C, de Andrade DOV, Brischoux F, Carretero MA, Dupoué A, Gavira RS, Lourdais O, Sannolo M, Van Dooren TJ (2021b) A worldwide and annotated database of evaporative water loss rates in squamate reptiles. Glob Ecol Biogeogr 30: 1938–1950. 10.1111/geb.13355.

[ref170] Le Galliard JF, Rozen-Rechels D, Lecomte A, Demay C, Dupoué A, Meylan S (2021a) Short-term changes in air humidity and water availability weakly constrain thermoregulation in a dry-skinned ectotherm. PloS One 16: e0247514. 10.1371/journal.pone.0247514. 33635881 PMC7909639

[ref171] Legendre LJ, Choi S, Clarke JA (2022) The diverse terminology of reptile eggshell microstructure and its effect on phylogenetic comparative analyses. J Anat 241: 641–666. 10.1111/joa.13723. 35758681 PMC9358755

[ref172] Lemenager LA, Tracy CR, Christian KA, Tracy CR (2022) Physiological control of water exchange in anurans. Ecol Evol 12: e8597. 10.1002/ece3.8597. 35169455 PMC8831224

[ref173] Lertzman-Lepofsky GF, Kissel AM, Sinervo B, Palen WJ (2020) Water loss and temperature interact to compound amphibian vulnerability to climate change. Glob Chang Biol 26: 4868–4879. 10.1111/gcb.15231. 32662211

[ref174] Li J, Wang X, Lan T, Lu Y, Hong M, Ding L, Wang L (2022) CDK5/NFAT5-regulated transporters involved in osmoregulation in *Fejervarya cancrivora*. Biology 11: 858. 10.3390/biology11060858. 35741379 PMC9220195

[ref175] Lieth H (1973) Primary production: terrestrial ecosystems. Hum Ecol 1: 303–332. 10.1007/BF01536729.

[ref176] Lighton J, Halsey L (2011) Flow-through respirometry applied to chamber systems: pros and cons, hints and tips. Comp Biochem Physiol Part A Mol Integr Physiol 158: 265–275. 10.1016/j.cbpa.2010.11.026. 21134483

[ref177] Lighton JRB (2019) Measuring Metabolic Rates: A Manual for Scientists. Oxford University Press, New York

[ref178] Lillywhite H, Menon J, Menon G, Sheehy C 3rd, Tu M-C (2009) Water exchange and permeability properties of the skin in three species of amphibious sea snakes (*Laticauda* spp.). J Exp Biol 212: 1921–1929. 10.1242/jeb.028704. 19483010

[ref179] Lillywhite HB (2006) Water relations of tetrapod integument. J Exp Biol 209: 202–226. 10.1242/jeb.02007. 16391344

[ref180] Lillywhite, H. B. (2016). Behavior and physiology: an ecological and evolutionary viewpoint on the energy and water relations of ectothermic amphibians and reptiles. In Amphibian and Reptile Adaptations to the Environment, eds. D. V. Andrade, C. R. Bevier and J. E. de Carvalho), pp. 1–40. Boca Raton, US: CRC Press, 10.1201/b20420-1.

[ref181] Lorrain-Soligon L, Robin F, Brischoux F (2022) Hydric status influences salinity-dependent water selection in frogs from coastal wetlands. Physiol Behav 249: 113775. 10.1016/j.physbeh.2022.113775. 35259400

[ref182] Loughland I, Little A, Seebacher F (2021) DNA methyltransferase 3a mediates developmental thermal plasticity. BMC Biol 19: 1–11. 10.1186/s12915-020-00942-w. 33478487 PMC7819298

[ref183] Lourdais O, Brischoux F, Barantin L (2005) How to assess musculature and performance in a constricting snake? A case study in the Colombian rainbow boa (*Epicrates cenchria maurus)*. J Zool 265: 43–51. 10.1017/S095283690400603X.

[ref184] Lourdais O, Dupoué A, Guillon M, Guiller G, Michaud B, DeNardo DF (2017) Hydric “costs” of reproduction: pregnancy increases evaporative water loss in the snake *Vipera aspis*. Physiol Biochem Zool 90: 663–672. 10.1086/694848. 29068263

[ref185] Lourdais O, Gartner GE, Brischoux F (2014) Ambush or active life: foraging mode influences haematocrit levels in snakes. Biol J Linn Soc 111: 636–645. 10.1111/bij.12223.

[ref186] Lourdais O, Hoffman TC, DeNardo DF (2007) Maternal brooding in the Children’s python (*Antaresia childreni*) promotes egg water balance. J Comp Physiol B 177: 569–577. 10.1007/s00360-007-0155-6. 17390138

[ref187] Lourdais O, Lorioux S, Dupoué A, Wright C, DeNardo DF (2015) Embryonic water uptake during pregnancy is stage-and fecundity-dependent in the snake *Vipera aspis*. Comp. Biochem. Physiol. Part A Mol. Integr. Physiol. 189: 102–106. 10.1016/j.cbpa.2015.07.019. 26255703

[ref188] Maclean IM, Klinges DH (2021) Microclimc: a mechanistic model of above, below and within-canopy microclimate. Ecol Model 451: 109567. 10.1016/j.ecolmodel.2021.109567.

[ref189] Madelaire CB, Barsotti AM, Wagener C, Vieira Sugano YY, Baxter-Gilbert J, Gomes FR, Measey J (2020) Challenges of dehydration result in a behavioral shift in invasive toads. Behav Ecol Sociobiol 74: 83. 10.1007/s00265-020-02866-5.

[ref190] Madliger CL, Love OP, Hultine KR, Cooke SJ (2018) The conservation physiology toolbox: status and opportunities. Conserv Physiol 6: coy029. 10.1093/conphys/coy029. 29942517 PMC6007632

[ref191] Madsen T, Loman J, Bauwens D, Stille B, Anderberg H, Anderberg L, Ujvari B (2023) The impact of an extreme climatic event on adder (*Vipera berus*) demography in southern Sweden. Biol J Linn Soc 138: 282–288. 10.1093/biolinnean/blac147.

[ref192] Maia MR (2014) Intra- and Interspecific Variation in Microhábitat Selection and its Relevance to the Maintenance of Water Balance in Anurans. MSc. University of São Paulo, SP, Brasil.

[ref193] Malik AI, Storey JM, Storey KB (2023) Regulation of the unfolded protein response during dehydration stress in African clawed frogs. Xenopus laevis Cell Stress Chaperon 28: 529–540. 10.1007/s12192-022-01275-z. PMC10468459 35484355

[ref194] Malik AI, Storey KB (2009) Activation of antioxidant defense during dehydration stress in the African clawed frog. Gene 442: 99–107. 10.1016/j.gene.2009.04.007. 19379800

[ref195] Malvin GM, Wood SC (1991) Behavioral thermoregulation of the toad, *Bufo marinus*: effects of air humidity. J Exp Zool 258: 322–326. 10.1002/jez.1402580307. 1909745

[ref196] Marco A, Díaz-Paniagua C (2008) Aggregation protects flexible-shelled reptile eggs from severe hydric stress. J Comp Physiol B 178: 421–428. 10.1007/s00360-007-0234-8. 18074142

[ref197] Márquez-García M, Correa-Solís M, Méndez M (2010) Life-history trait variation in tadpoles of the warty toad in response to pond drying. J Zool 281: 105–111. 10.1111/j.1469-7998.2009.00684.x.

[ref198] Marra NJ, Eo SH, Hale MC, Waser PM, DeWoody JA (2012) A priori and a posteriori approaches for finding genes of evolutionary interest in non-model species: osmoregulatory genes in the kidney transcriptome of the desert rodent *Dipodomys spectabilis* (banner-tailed kangaroo rat). Comp Biochem Physiol Part D Genomics Proteomics 7: 328–339. 10.1016/j.cbd.2012.07.001. 22841684

[ref199] Martins F, Kruuk L, Llewelyn J, Moritz C, Phillips B (2019) Heritability of climate-relevant traits in a rainforest skink. Heredity 122: 41–52. 10.1038/s41437-018-0085-y. 29789644 PMC6288132

[ref200] Mathwin R, Wassens S, Gibbs MS, Young J, Ye Q, Saltré F, Bradshaw CJ (2023) Modeling the effects of water regulation on the population viability of a threatened amphibian. Ecosphere 14: e4379. 10.1002/ecs2.4379.

[ref201] Mathwin R, Wassens S, Young J, Ye Q, Bradshaw CJ (2021) Manipulating water for amphibian conservation. Conserv Biol 35: 24–34. 10.1111/cobi.13501. 32189374

[ref202] Mausbach J, Laurila A, Räsänen K (2022) Context dependent variation in corticosterone and phenotypic divergence of *Rana arvalis* populations along an acidification gradient. BMC Ecology and Evolution 22: 11. 10.1186/s12862-022-01967-1. 35123416 PMC8818180

[ref203] Mautz WJ (1980) Factors influencing evaporative water loss in lizards. Comp. Biochem. Physiol. Part A Physiol. 67: 429–437. 10.1016/S0300-9629(80)80019-3.

[ref204] McClanahan L Jr, Baldwin R (1969) Rate of water uptake through the integument of the desert toad, *Bufo punctatus*. Comp Biochem Physiol 28: 381–389. 10.1016/0010-406X(69)91351-6. 5777385

[ref205] McCormick SD, Bradshaw D (2006) Hormonal control of salt and water balance in vertebrates. Gen Comp Endocrinol 147: 3–8. 10.1016/j.ygcen.2005.12.009. 16457828

[ref206] McKechnie AE, Wolf BO (2019) The physiology of heat tolerance in small endotherms. Phys 34: 302–313. 10.1152/physiol.00011.2019. 31389778

[ref207] McKee TB, Doesken NJ, Kleist J (1993) The relationship of drought frequency and duration to time scales. In Proceedings of the 8th Conference on Applied Climatology 17, California, USA, pp. 179–183.

[ref208] McTernan MR, Sears MW (2022) Repeatability of voluntary thermal maximum and covariance with water loss reveal potential for adaptation to changing climates. Physiol Biochem Zool 95: 113–121. 10.1086/717938. 34986078

[ref209] Meyer AV, Sakairi Y, Kearney MR, Buckley LB (2023) A guide and tools for selecting and accessing microclimate data for mechanistic niche modeling. Ecosphere 14: e4506. 10.1002/ecs2.4506.

[ref210] Millikin AR, Woodley SK, Davis DR, Moore IT, Anderson JT (2019) Water-borne and plasma corticosterone are not correlated in spotted salamanders. Ecol Evol 9: 13942–13953. 10.1002/ece3.5831. 31938493 PMC6953692

[ref211] Minter NJ, Buatois LA, Mángano MG, Davies NS, Gibling MR, MacNaughton RB, Labandeira CC (2017) Early bursts of diversification defined the faunal colonization of land. Nat Eco Evol 1: 0175. 10.1038/s41559-017-0175.

[ref212] Mitchell A, Bergmann PJ (2016) Thermal and moisture habitat preferences do not maximize jumping performance in frogs. Funct. Ecol. 30: 733–742. 10.1111/1365-2435.12535.

[ref213] Mitchell D, Snelling EP, Hetem RS, Maloney SK, Strauss WM, Fuller A (2018) Revisiting concepts of thermal physiology: predicting responses of mammals to climate change. J Anim Ecol 87: 956–973. 10.1111/1365-2656.12818. 29479693

[ref214] Mitchell NJ (2001) Males call more from wetter nests: effects of substrate water potential on reproductive behaviours of terrestrial toadlets. Proc R Soc B Biol Sci 268: 87–93. 10.1098/rspb.2000.1334. PMC1087605 12123303

[ref215] Moeller KT, Demare G, Davies S, DeNardo DF (2017) Dehydration enhances multiple physiological defense mechanisms in a desert lizard, *Heloderma suspectum*. J Exp Biol 220: 2166–2174. 10.1242/jeb.150367. 28432151

[ref216] Moen DS, Cabrera-Guzmán E, Caviedes-Solis IW, González-Bernal E, Hanna AR (2022) Phylogenetic analysis of adaptation in comparative physiology and biomechanics: overview and a case study of thermal physiology in treefrogs. J Exp Biol 225: jeb243292. 10.1242/jeb.243292. 35119071

[ref217] Moen DS, Winne CT, Reed RN (2005) Habitat-mediated shifts and plasticity in the evaporative water loss rates of two congeneric pit vipers (Squamata, Viperidae, Agkistrodon). Evol Ecol Res 7: 759–766.

[ref218] Monaghan P (2008) Early growth conditions, phenotypic development and environmental change. Philos Trans R Soc B Biol Sci 363: 1635–1645. 10.1098/rstb.2007.0011. PMC2606729 18048301

[ref219] Moore D, Stow A, Kearney MR (2018) Under the weather?—The direct effects of climate warming on a threatened desert lizard are mediated by their activity phase and burrow system. J Anim Ecol 87: 660–671. 10.1111/1365-2656.12812. 29446081

[ref220] Moreira DC, Carvajalino-Fernandez JM, Silva WP, Kuzniewski F, Navas CA, de Carvalho JE, Hermes-Lima M (2020) Preparation for oxidative stress in *Proceratophrys cristicep*s (Anura, Odontophrynidae) naturally estivating in the Brazilian Caatinga. Sci Total Environ 723: 137957. 10.1016/j.scitotenv.2020.137957. 32220732

[ref221] Moretti M, Dias AT, De Bello F, Altermatt F, Chown SL, Azcárate FM, Bell JR, Fournier B, Hedde M, Hortal J (2017) Handbook of protocols for standardized measurement of terrestrial invertebrate functional traits. Funct. Ecol. 31: 558–567. 10.1111/1365-2435.12776.

[ref222] Morgan R, Finnøen MH, Jensen H, Pélabon C, Jutfelt F (2020) Low potential for evolutionary rescue from climate change in a tropical fish. Proc Natl Acad Sci 117: 33365–33372. 10.1073/pnas.2011419117. 33318195 PMC7776906

[ref223] Moss WE, Crausbay SD, Rangwala I, Wason JW, Trauernicht C, Stevens-Rumann CS, Sala A, Rottler CM, Pederson GT, Miller BW et al. (2024) Drought as an emergent driver of ecological transformation in the twenty-first century. Bioscience 74: 524–538. 10.1093/biosci/biae050. 39872081 PMC11770345

[ref224] Motoshima T, Nagashima A, Ota C, Oka H, Hosono K, Braasch I, Nishihara H, Kato A (2023) Na+/Cl− cotransporter 2 is not fish-specific and is widely found in amphibians, non-avian reptiles, and select mammals. Physiol Genomics 55: 113–131. 10.1152/physiolgenomics.00143.2022. 36645671 PMC9988527

[ref225] Moustakis Y, Papalexiou SM, Onof CJ, Paschalis A (2021) Seasonality, intensity, and duration of rainfall extremes change in a warmer climate. Earth's Future 9: e2020EF001824. 10.1029/2020EF001824.

[ref226] Nagy, K. (1989). Doubly-labeled water studies of vertebrate physiological ecology. In Stable Isotopes in Ecological Research, eds. P. W. Rundel, J. R. Ehleringer and K. A. Nagy), pp. 270–287: Springer, London, UK. 10.1007/978-1-4612-3498-2_16.

[ref227] Nagy KA (1980) CO2 production in animals: analysis of potential errors in the doubly labeled water method. Am J Physiol-Reg I 238: R466–R473. 10.1152/ajpregu.1980.238.5.R466. 6769345

[ref228] Nagy KA, Medica PA (1986) Physiological ecology of desert tortoises in southern Nevada. Herpetologica 42: 73–92.

[ref229] Nath RK, Deb S (2010) Water-body area extraction from high resolution satellite images-an introduction, review, and comparison. Int J Imag Process 3: 265–384.

[ref230] Navas, C. A. and Carvalho, J. E. (2010). Aestivation: Molecular and Physiological Aspects. Berlin, Germany: Springer, 10.1007/978-3-642-02421-4.

[ref231] Navas CA, Jared C, Antoniazzi MM (2002) Water economy in the casque-headed tree-frog *Corythomantis greeningi* (Hylidae): role of behaviour, skin, and skull skin co-ossification. J Zool 257: 525–532. 10.1017/S0952836902001103.

[ref232] Nevarez JG, Acierno MJ, Angel M, Beaufrere H (2012) Determination of agreement between measured and calculated plasma osmolality values in captive-reared American alligators (*Alligator mississippiensis*). J Herpetol Med Surg 22: 36–41. 10.5818/1529-9651-22.1-2.36.

[ref233] Nevo E (1973) Adaptive variation in size of cricket frogs. Ecology 54: 1271–1281. 10.2307/1934189.

[ref234] Newman RA (1988) Adaptive plasticity in development of *Scaphiopus couchii* tadpoles in desert ponds. Evolution 42: 774–783. 10.2307/2408868. 28563867

[ref235] Nolan N, Hayward M, Callen A, Klop-Toker K (2025) Hydroperiod influences tadpole growth and development in the endangered Littlejohn's tree frog (*Litoria littlejohni*). Ecol Evol 15: e70829. 10.1002/ece3.70829. 39803208 PMC11717664

[ref236] Northrop V (2024) Olfactory Navigation to Water Resources and Deferred Intake of Brackish Water During Dehydration in a Xeric-Adapted Species, the Gila Monster (Heloderma suspectum). School of Life Sciences, Arizona State University, MSc

[ref237] Novick KA, Ficklin DL, Grossiord C, Konings AG, Martínez-Vilalta J, Sadok W, Trugman AT, Williams AP, Wright AJ, Abatzoglou JT et al. (2024) The impacts of rising vapour pressure deficit in natural and managed ecosystems. Plant Cell Environ 47: 3561–3589. 10.1111/pce.14846. 38348610

[ref238] Ohmer ME, Hammond TT, Switzer S, Wantman T, Bednark JG, Paciotta E, Coscia J, Richards-Zawacki CL (2023) Developmental environment has lasting effects on amphibian post-metamorphic behavior and thermal physiology. J Exp Biol 226: jeb244883. 10.1242/jeb.244883. 37039737

[ref239] Oki T, Kanae S (2006) Global hydrological cycles and world water resources. Science 313: 1068–1072. 10.1126/science.1128845. 16931749

[ref240] Ouellet S, Lavictoire A, Laberge F (2020) Determinants of the water seeking response in a T-maze in the fire-bellied toad *Bombina orientalis*. Learn Motiv 72: 101679. 10.1016/j.lmot.2020.101679.

[ref241] Oufiero CE, Van Sant MJ (2018) Variation and repeatability of cutaneous water loss and skin resistance in relation to temperature and diel variation in the lizard *Sceloporus consobrinus*. J Comp Physiol B 188: 671–681. 10.1007/s00360-018-1156-3. 29619510

[ref242] Owen JG (1989) Patterns of herpetofaunal species richness: relation to temperature, precipitation, and variance in elevation. J Biogeogr 16: 141–150. 10.2307/2845088.

[ref243] Palmer WC (1965) In UW Bureau, ed, Meteorological Drought Vol vol. 45, US Weather Bureau, Washington, DC, pp. 1–58.

[ref244] Perry SM, Acierno MJ, Mitchell MA (2020) Measuring the level of agreement between osmometer and calculated plasma osmolalities in two species of chameleons, *Furcifer pardalis* and *Chamaeleo calyptratus*. J. Herpetol. Med. Surg. 31: 36–42. 10.5818/11-2020.

[ref245] Peterson, A. T., Soberón, J., Pearson, R. G., Anderson, R. P., Martínez-Meyer, E., Nakamura M. and Araújo, M. B. (2011). Ecological Niches and Geographic Distributions. US: Princeton University Press, 10.23943/princeton/9780691136868.001.0001.

[ref246] Pettersen AK, Metcalfe NB, Seebacher F (2024) Intergenerational plasticity aligns with temperature-dependent selection on offspring metabolic rates. Philos Trans R Soc B 379: 20220496. 10.1098/rstb.2022.0496. PMC10772613 38186279

[ref247] Pincheira-Donoso D, Meiri S, Jara M, Olalla-Tárraga MÁ, Hodgson DJ (2019) Global patterns of body size evolution are driven by precipitation in legless amphibians. Ecography 42: 1682–1690. 10.1111/ecog.04644.

[ref248] Pintor AF, Schwarzkopf L, Krockenberger AK (2016) Hydroregulation in a tropical dry-skinned ectotherm. Oecologia 182: 925–931. 10.1007/s00442-016-3687-1. 27384338

[ref249] Pirtle, E. I., Tracy, C. R. and Kearney, M. R. (2019). Hydroregulation: a neglected behavioral response of lizards to climate change? In Behavior of Lizards, eds. V. Bels and A. Russell), pp. 343–374: CRC Press, Florida, USA. 10.1201/9781498782739-12.

[ref250] Pisor AC, Touma D, Singh D, Jones JH (2023) To understand climate change adaptation, we must characterize climate variability: here’s how. One Earth 6: 1665–1676. 10.1016/j.oneear.2023.11.005.

[ref251] Porter W, Mitchell J, Beckman W, DeWitt C (1973) Behavioral implications of mechanistic ecology. Oecologia 13: 1–54. 10.1007/BF00379617. 28307981

[ref252] Pottier P, Kearney MR, Wu NC, Gunderson AR, Reij JE, Reivera-Villanueva N, Pollo P, Burke S, Drobniak SM, Nakagawa S (2025) Vulnerability of amphibians to global warming. Nature 639: 954–961. 10.1038/s41586-025-08665-0. 40044855 PMC11946914

[ref253] Pough FH, Gans C (1982) Biology of the Reptilia, Volume 12, Physiology C: Physiological Ecology. Academic Press, London.

[ref254] Pough FH, Taigen TL, Stewart MM, Brussard PF (1983) Behavioral modification of evaporative water loss by a Puerto Rican frog. Ecology 64: 244–252. 10.2307/1937072.

[ref255] Reemeyer JE, Rumball D, Mandrak NE, Chapman LJ (2024) Seasonal variation in thermal tolerance and hypoxia tolerance of a threatened minnow and a non-imperilled congener: a cautionary tale for surrogate species in conservation. Conserv Physiol 12: coae071. 10.1093/conphys/coae071. 39417164 PMC11482009

[ref256] Repp RA, Schuett GW (2008) Western diamond-backed rattlesnakes, *Crotalus atrox* (Serpentes: Viperidae), gain water by harvesting and drinking rain, sleet, and snow. Southwest Nat 53: 108–114. 10.1894/0038-4909(2008)53[108:WDRCAS]2.0.CO;2.

[ref257] Reshetnikov AN (1998) Searching for water by the common treefrog (*Hyla arborea*) and the green toad (*Bufo viridis*): the perception of odours or air humidity. In SL Kuzmin, CK Dodd, eds, Advances in Amphibian Research in the Former Soviet Union Vol 1. Pensoft Publishing, Sofia, Bulgaria, pp. 105–112.

[ref258] Ribou A-C (2016) Synthetic sensors for reactive oxygen species detection and quantification: a critical review of current methods. Antioxid Redox Signal 25: 520–533. 10.1089/ars.2016.6741. 27225539

[ref259] Richter-Boix A, Tejedo M, Rezende EL (2011) Evolution and plasticity of anuran larval development in response to desiccation. A comparative analysis. Ecol Evol 1: 15–25. 10.1002/ece3.2. 22393479 PMC3287374

[ref260] Riddell E, Iknayan K, Hargrove L, Tremor S, Patton J, Ramirez R, Wolf B, Beissinger S (2021) Exposure to climate change drives stability or collapse of desert mammal and bird communities. Science 371: 633–636. 10.1126/science.abd4605. 33542137

[ref261] Riddell EA, Apanovitch EK, Odom JP, Sears MW (2017) Physical calculations of resistance to water loss improve predictions of species range models. Ecological monographs 87: 21–33. 10.1002/ecm.1240.

[ref262] Riddell EA, Burger IJ, Muñoz MM, Weaver SJ, Womack MC (2024) Amphibians exhibit extremely high hydric costs of respiration. Integr Comp Biol 64: 366–376. 10.1093/icb/icae053. 38802122

[ref263] Riddell EA, McPhail J, Damm JD, Sears MW (2018a) Trade-offs between water loss and gas exchange influence habitat suitability of a woodland salamander. Funct. Ecol. 32: 916–925. 10.1111/1365-2435.13030.

[ref264] Riddell EA, Odom JP, Damm JD, Sears MW (2018b) Plasticity reveals hidden resistance to extinction under climate change in the global hotspot of salamander diversity. Sci Adv 4: eaar5471. 10.1126/sciadv.aar5471. 30014037 PMC6047487

[ref265] Riddell EA, Roback EY, Wells CE, Zamudio KR, Sears MW (2019) Thermal cues drive plasticity of desiccation resistance in montane salamanders with implications for climate change. Nat Commun 10: 4091. 10.1038/s41467-019-11990-4. 31501425 PMC6733842

[ref266] Ritchie DJ, Friesen CR (2022) Invited review: thermal effects on oxidative stress in vertebrate ectotherms. Comp. Biochem. Physiol. Part A Mol. Integr. Physiol. 263: 111082. 10.1016/j.cbpa.2021.111082. 34571153

[ref267] Roberts JB, Lillywhite HB (1983) Lipids and the permeability of epidermis from snakes. J Exp Zool 228: 1–9. 10.1002/jez.1402280102.

[ref268] Roe JH, Georges A, Green B (2008) Energy and water flux during terrestrial estivation and overland movement in a freshwater turtle. Physiol Biochem Zool 81: 570–583. 10.1086/589840. 18717626

[ref269] Rosenzweig ML (1968) Net primary productivity of terrestrial communities: prediction from climatological data. Am Nat 102: 67–74. 10.1086/282523.

[ref270] Rouse JW Jr, Haas RH, Deering D, Schell J, Harlan JC (1974) Monitoring the vernal advancement and retrogradation (green wave effect) of natural vegetation. Texas A&M University, Texas.

[ref271] Rozen-Rechels D, Badiane A, Agostini S, Meylan S, Le Galliard JF (2020b) Water restriction induces behavioral fight but impairs thermoregulation in a dry-skinned ectotherm. Oikos 129: 572–584. 10.1111/oik.06910.

[ref272] Rozen-Rechels D, Dupoué A, Lourdais O, Chamaillé-Jammes S, Meylan S, Clobert J, Le Galliard JF (2019) When water interacts with temperature: ecological and evolutionary implications of thermo-hydroregulation in terrestrial ectotherms. Ecol Evol 9: 10029–10043. 10.1002/ece3.5440. 31534711 PMC6745666

[ref273] Rozen-Rechels D, Dupoué A, Meylan S, Qitout K, Decencière B, Agostini S, Le Galliard JF (2020a) Acclimation to water restriction implies different paces for behavioral and physiological responses in a lizard species. Physiol Biochem Zool 93: 160–174. 10.1086/707409. 32031477

[ref274] Ruthsatz K, Dausmann KH, Drees C, Becker LI, Hartmann L, Reese J, Reinhardt S, Robinson T, Sabatino NM, Peck MA et al. (2020) Altered thyroid hormone levels affect the capacity for temperature-induced developmental plasticity in larvae of *Rana temporaria* and *Xenopus laevis*. J Therm Biol 90: 102599. 10.1016/j.jtherbio.2020.102599. 32479394

[ref275] Ruthsatz K, Eterovick PC, Bartels F, Mausbach J (2023b) Contributions of water-borne corticosterone as one non-invasive biomarker in assessing nitrate pollution stress in tadpoles of *Rana temporaria*. Gen Comp Endocrinol 331: 114164. 10.1016/j.ygcen.2022.114164. 36400158

[ref276] Ruthsatz K, Rico-Millan R, Eterovick PC, Gomez-Mestre I (2023a) Exploring water-borne corticosterone collection as a non-invasive tool in amphibian conservation physiology: benefits, limitations and future perspectives. Conserv. Physiol. 11: coad070. 10.1093/conphys/coad070. 37663928 PMC10472495

[ref277] Salazar JC, Miles DB (2024) The shape of water: physiological adaptations to habitat aridity in the ornate tree lizard (*Urosaurus ornatus*). Integr. Comp. Biol. 64: 390–401. 10.1093/icb/icae061. 38844405

[ref278] Sandfoss MR, Lillywhite HB (2019) Water relations of an insular pit viper. J Exp Biol 222: jeb204065. 10.1242/jeb.204065. 30975741

[ref279] Sawyer WH, Schisgall RM (1956) Increased permeability of the frog bladder to water in response to dehydration and neurohypophysial extracts. American Journal of Physiology-Legacy Content 187: 312–314. 10.1152/ajplegacy.1956.187.2.312. 13372783

[ref280] Schmuck R, Linsenmair K (1997) Regulation of body water balance in reedfrogs (superspecies *Hyperolius viridiflavus* and *Hyperolius marmoratus*: Amphibia, Anura, Hyperoliidae) living in unpredictably varying savannah environments. Comp Biochem Physiol Part A Physiol 118: 1335–1352. 10.1016/S0300-9629(97)86804-1. 9505437

[ref281] Scholz RW, Blumer YB, Brand FS (2012) Risk, vulnerability, robustness, and resilience from a decision-theoretic perspective. Journal of Risk Research 15: 313–330. 10.1080/13669877.2011.634522.

[ref282] Seebacher F, White CR, Franklin CE (2015) Physiological plasticity increases resilience of ectothermic animals to climate change. Nat Clim Change 5: 61–66. 10.1038/nclimate2457.

[ref283] Senior AF, Atkins ZS, Clemann N, Gardner MG, Schroder M, While GM, Wong BB, Chapple DG (2019) Variation in thermal biology of three closely related lizard species along an elevation gradient. Biol J Linn Soc 127: 278–291. 10.1093/biolinnean/blz046.

[ref284] Senzano LM, Andrade DV (2018) Temperature and dehydration effects on metabolism, water uptake and the partitioning between respiratory and cutaneous evaporative water loss in a terrestrial toad. J Exp Biol 221: jeb188482. 10.1242/jeb.188482. 30385484

[ref285] Senzano LM, Bovo RP, Andrade DV (2022) Empirical estimation of skin resistance to water loss in amphibians: agar evaluation as a non-resistance model to evaporation. J Exp Biol 225: jeb243941. 10.1242/jeb.243941. 35818822

[ref286] Serrano-Rojas SJ, Pašukonis A (2021) Tadpole-transporting frogs use stagnant water odor to find pools in the rainforest. J Exp Biol 224: jeb243122. 10.1242/jeb.243122. 34608492 PMC8627569

[ref287] Servino LM, Verdade VK, Sawaya RJ (2022) For neither heat nor water conservation: body size variation in Atlantic Forest frogs does not follow a general mechanism. J Biogeogr 49: 460–468. 10.1111/jbi.14309.

[ref288] Seymour RS, Kennett R, Christian K (1997) Osmotic balance in the eggs of the turtle *Chelodina rugosa* during developmental arrest under water. Physiol Zool 70: 301–306. 10.1086/639604. 9231404

[ref289] Shama LN, Wegner KM (2014) Grandparental effects in marine sticklebacks: transgenerational plasticity across multiple generations. J Evol Biol 27: 2297–2307. 10.1111/jeb.12490. 25264208

[ref290] Sherbrooke WC (1993) Rain-drinking behaviors of the Australian thorny devil (Sauria: Agamidae). J Herpetol 27: 270–275. 10.2307/1565147.

[ref291] Sheridan JA, Mendenhall CD, Yambun P (2022) Frog body size responses to precipitation shift from resource-driven to desiccation-resistant as temperatures warm. Ecol Evol 12: e9589. 10.1002/ece3.9589. 36523519 PMC9745258

[ref292] Shibata Y, Sano T, Tsuchiya N, Okada R, Mochida H, Tanaka S, Suzuki M (2014) Gene expression and localization of two types of AQP5 in *Xenopus tropicalis* under hydration and dehydration. Am J Physiol-Reg I 307: R44–R56. 10.1152/ajpregu.00186.2013 24717674

[ref293] da Silveira Scarpellini C, Bícego KC, Tattersall GJ (2015) Thermoregulatory consequences of salt loading in the lizard *Pogona vitticeps*. J Exp Biol 218: 1166–1174. 10.1242/jeb.116723. 25714566

[ref294] Sinai N, Glos J, Mohan AV, Lyra ML, Riepe M, Thöle E, Zummach C, Ruthsatz K (2022) Developmental plasticity in amphibian larvae across the world: investigating the roles of temperature and latitude. J Therm Biol 106: 103233. 10.1016/j.jtherbio.2022.103233. 35636893

[ref295] Sinclair BJ, Saruhashi S, Terblanche JS (2024) Integrating water balance mechanisms into predictions of insect responses to climate change. J Exp Biol 227: jeb247167. 10.1242/jeb.247167. 38779934

[ref296] Sinervo B, Mendez-De-La-Cruz F, Miles DB, Heulin B, Bastiaans E, Villagrán-Santa Cruz M, Lara-Resendiz R, Martínez-Méndez N, Calderón-Espinosa ML, Meza-Lázaro RN (2010) Erosion of lizard diversity by climate change and altered thermal niches. Science 328: 894–899. 10.1126/science.1184695. 20466932

[ref297] Somero G (2010) The physiology of climate change: how potentials for acclimatization and genetic adaptation will determine ‘winners’ and ‘losers’. J Exp Biol 213: 912–920. 10.1242/jeb.037473. 20190116

[ref298] Souza KS, Fortunato DS, Jardim L, Terribile LC, Lima-Ribeiro MS, Mariano CÁ, Pinto-Ledezma JN, Loyola R, Dobrovolski R, Rangel TF et al. (2023) Evolutionary rescue and geographic range shifts under climate change for global amphibians. Front Ecol Evol 11: 1038018. 10.3389/fevo.2023.1038018.

[ref299] Spence C, Mengistu S (2016) Deployment of an unmanned aerial system to assist in mapping an intermittent stream. Hydrol Process 30: 493–500. 10.1002/hyp.10597.

[ref300] Spotila JR, Berman EN (1976) Determination of skin resistance and the role of the skin in controlling water loss in amphibians and reptiles. Comp Biochem Physiol Part A Physiol 55: 407–411. 10.1016/0300-9629(76)90069-4. 9259

[ref301] Stahlschmidt Z, DeNardo DF (2010) Parental behavior in pythons is responsive to both the hydric and thermal dynamics of the nest. J Exp Biol 213: 1691–1696. 10.1242/jeb.041095. 20435820

[ref302] Stahlschmidt ZR, Hoffman TC, DeNardo DF (2008) Postural shifts during egg-brooding and their impact on egg water balance in Children’s pythons (*Antaresia childreni*). Ethology 114: 1113–1121. 10.1111/j.1439-0310.2008.01553.x.

[ref303] Stanley TR, Clark RW, Fisher RN, Rochester CJ, Root SA, Lombardo KJ, Ostermann-Kelm SD (2020) Changes in capture rates and body size among vertebrate species occupying an insular urban habitat reserve. Conserv Sci Pract 2: e245. 10.1111/csp2.245.

[ref304] Suzuki M, Shibata Y, Ogushi Y, Okada R (2015) Molecular machinery for vasotocin-dependent transepithelial water movement in amphibians: aquaporins and evolution. Biol Bull 229: 109–119. 10.1086/BBLv229n1p109. 26338873

[ref305] Suzuki M, Tanaka S (2009) Molecular and cellular regulation of water homeostasis in anuran amphibians by aquaporins. Comp Biochem Physiol Part A Mol Integr Physiol 153: 231–241. 10.1016/j.cbpa.2009.02.035. 19268556

[ref306] Székely D, Cogălniceanu D, Székely P, Denoël M (2018) Dryness affects burrowing depth in a semi-fossorial amphibian. J Arid Environ 155: 79–81. 10.1016/j.jaridenv.2018.02.003.

[ref307] Székely D, Denoël M, Székely P, Cogălniceanu D (2017) Pond drying cues and their effects on growth and metamorphosis in a fast developing amphibian. J Zool 303: 129–135. 10.1111/jzo.12468.

[ref308] Taigen TL, Pough FH, Stewart MM (1984) Water balance of terrestrial anuran (*Eleutherodactylus coqui*) eggs: importance of parental care. Ecology 65: 248–255. 10.2307/1939477.

[ref309] Tattersall GJ, Eterovick PC, de Andrade DV (2006) Tribute to RG Boutilier: skin colour and body temperature changes in basking *Bokermannohyla alvarengai* (Bokermann 1956). J Exp Biol 209: 1185–1196. 10.1242/jeb.02038. 16547291

[ref310] Taylor EN, Diele-Viegas LM, Gangloff EJ, Hall JM, Halpern B, Massey MD, Rödder D, Rollinson N, Spears S, Sun B et al. (2020) The thermal ecology and physiology of reptiles and amphibians: a user's guide. J Exp Zool Part A 335: 13–44. 10.1002/jez.2396. 32638552

[ref311] Theissinger K, Fernandes C, Formenti G, Bista I, Berg PR, Bleidorn C, Bombarely A, Crottini A, Gallo GR, Godoy JA et al. (2023) How genomics can help biodiversity conservation. Trends Genet 39: 545–559. 10.1016/j.tig.2023.01.005. 36801111

[ref312] Thompson CM, Popescu VD (2021) Complex hydroperiod induced carryover responses for survival, growth, and endurance of a pond-breeding amphibian. Oecologia 195: 1071–1081. 10.1007/s00442-021-04881-3. 33635404

[ref313] Thompson MM, Rowley JJ, Poore AG, Callaghan CT (2022) Citizen science reveals meteorological determinants of frog calling at a continental scale. Divers Distrib 28: 2375–2387. 10.1111/ddi.13634.

[ref314] Thorson TB (1955) The relationship of water economy to terrestrialism in amphibians. Ecology 36: 100–116. 10.2307/1931435.

[ref315] Thorson TB (1968) Body fluid partitioning in Reptilia. Copeia 1968: 592–601. 10.2307/1442030.

[ref316] Tingley R, Greenlees M, Shine R (2012) Hydric balance and locomotor performance of an anuran (*Rhinella marina*) invading the Australian arid zone. Oikos 121: 1959–1965. 10.1111/j.1600-0706.2012.20422.x.

[ref317] Tollis M, DeNardo DF, Cornelius JA, Dolby GA, Edwards T, Henen BT, Karl AE, Murphy RW, Kusumi K (2017) The Agassiz’s desert tortoise genome provides a resource for the conservation of a threatened species. PloS One 12: e0177708. 10.1371/journal.pone.0177708. 28562605 PMC5451010

[ref318] Tracy CR (1976) A model of the dynamic exchanges of water and energy between a terrestrial amphibian and its environment. Ecological monographs 46: 293–326. 10.2307/1942256.

[ref319] Tracy CR, Christian KA, Betts G, Tracy CR (2008) Body temperature and resistance to evaporative water loss in tropical Australian frogs. Comp. Biochem. Physiol. Part A Mol. Integr. Physiol. 150: 102–108. 10.1016/j.cbpa.2006.04.031. 16829148

[ref320] Tracy CR, Laurence N, Christian KA (2011) Condensation onto the skin as a means for water gain by tree frogs in tropical Australia. Am Nat 178: 553–558. 10.1086/661908. 21956032

[ref321] Tracy CR, Tixier T, Le Nöene C, Christian KA (2014) Field hydration state varies among tropical frog species with different habitat use. Physiol Biochem Zool 87: 197–202. 10.1086/674537. 24642537

[ref322] Turner A, Heard G, Mathwin R, Bradshaw CJ, Hall A, Wassens S (2025) Cool-season environmental water delivery increases extinction risk for chytrid-infected amphibians. Conserv Sci Pract 7: e70022. 10.1111/csp2.70022.

[ref323] Uchiyama M, Konno N (2006) Hormonal regulation of ion and water transport in anuran amphibians. Gen Comp Endocrinol 147: 54–61. 10.1016/j.ygcen.2005.12.018. 16472810

[ref324] Urban MC, Bocedi G, Hendry AP, Mihoub J-B, Pe’er G, Singer A, Bridle J, Crozier L, De Meester L, Godsoe W (2016) Improving the forecast for biodiversity under climate change. Science 353: aad8466. 10.1126/science.aad8466. 27609898

[ref325] Vicenzi N, Bacigalupe LD, Laspiur A, Ibargüengoytía N, Sassi PL (2021) Could plasticity mediate highlands lizards’ resilience to climate change? A case study of the leopard iguana (*Diplolaemus leopardinus*) in Central Andes of Argentina. J Exp Biol 224: jeb242647. 10.1242/jeb.242647. 34160050

[ref326] Warner DA, Kelly C, Pruett JE, Fargevieille A, Klabacka RL (2023) Fluctuating environments hinder the ability of female lizards to choose suitable nest sites for their embryos. Behav Ecol Sociobiol 77: 32. 10.1007/s00265-023-03310-0.

[ref327] Wayne SM, Norris MC, Fargevieille A, Cobb K, Scruggs C, Miracle J, Herron V, Hall JM, Warner DA (2025) Reduced water availability to mothers and embryos has little effect on offspring phenotypes in an invasive lizard. J Exp Zool Part A 343: 535–545. 10.1002/jez.2906. 39888287

[ref328] Weaver SJ, Axsom IJ, Peria L, McIntyre T, Chung J, Telemeco RS, Westphal MF, Taylor EN (2024) Hydric physiology and ecology of a federally endangered desert lizard. Conserv. Physiol. 12: coae019. 10.1093/conphys/coae019. 38715929 PMC11074591

[ref329] Weaver SJ, Edwards H, McIntyre T, Temple SM, Alexander Q, Behrens MC, Biedebach RE, Budwal SS, Carlson JE, Castagnoli JO et al. (2022) Cutaneous evaporative water loss in lizards is variable across body regions and plastic in response to humidity. Herpetologica 78: 169–183. 10.1655/Herpetologica-D-21-00030.1.

[ref330] Weaver SJ, McIntyre T, van Rossum T, Telemeco RS, Taylor EN (2023) Hydration and evaporative water loss of lizards change in response to temperature and humidity acclimation. J Exp Biol 226: jeb246459. 10.1242/jeb.246459. 37767755

[ref331] Wells N, Goddard S, Hayes MJ (2004) A self-calibrating Palmer drought severity index. J Climate 17: 2335–2351. 10.1175/1520-0442(2004)017<2335:ASPDSI>2.0.CO;2.

[ref332] White CR, Marshall DJ, Chown SL, Clusella-Trullas S, Portugal SJ, Franklin CE, Seebacher F (2021) Geographical bias in physiological data limits predictions of global change impacts. Funct Ecol 35: 1572–1578. 10.1111/1365-2435.13807.

[ref333] Williams SE, Shoo LP, Isaac JL, Hoffmann AA, Langham G (2008) Towards an integrated framework for assessing the vulnerability of species to climate change. PLoS Biol 6: e325. 10.1371/journal.pbio.0060325. 19108608 PMC2605927

[ref334] Willumsen NJ, Viborg AL, Hillyard SD (2007) Vascular aspects of water uptake mechanisms in the toad skin: perfusion, diffusion, confusion. Comp. Biochem Physiol Part A Mol Integr Physiol 148: 55–63. 10.1016/j.cbpa.2006.12.032. 17331768

[ref335] Withers PC (1977) Measurement of VO2, VCO2, and evaporative water loss with a flow-through mask. J Appl Physiol 42: 120–123. 10.1152/jappl.1977.42.1.120. 833070

[ref336] Wolak ME, Fairbairn DJ, Paulsen YR (2012) Guidelines for estimating repeatability. Method. Ecol. Evol. 3: 129–137. 10.1111/j.2041-210X.2011.00125.x.

[ref337] Woodbury AM (1954) Study of reptile dens. Herpetologica 10: 49–53.

[ref338] Woodget AS, Austrums R, Maddock IP, Habit E (2017) Drones and digital photogrammetry: from classifications to continuums for monitoring river habitat and hydromorphology. Wiley Interdiscip Rev Water 4: e1222. 10.1002/wat2.1222.

[ref339] Woods HA, Smith JN (2010) Universal model for water costs of gas exchange by animals and plants. Proc Natl Acad Sci 107: 8469–8474. 10.1073/pnas.0905185107. 20404161 PMC2889562

[ref340] Word JM, Hillman SS (2005) Osmotically absorbed water preferentially enters the cutaneous capillaries of the pelvic patch in the toad *Bufo marinus*. Physiol Biochem Zool 78: 40–47. 10.1086/425196. 15702461

[ref341] Wright CD, Jackson ML, DeNardo DF (2013) Meal consumption is ineffective at maintaining or correcting water balance in a desert lizard, *Heloderma suspectum*. J Exp Biol 216: 1439–1447. 10.1242/jeb.080895. 23536591

[ref342] Wu C-S, Kam Y-C (2009) Effects of salinity on the survival, growth, development, and metamorphosis of *Fejervarya limnocharis* tadpoles living in brackish water. Zoolog Sci 26: 476–482. 10.2108/zsj.26.476. 19663642

[ref343] Wu NC, Alton LA, Bovo RP, Carey N, Currie SE, Lighton JR, McKechnie AE, Pottier P, Rossi G, White CR et al. (2024b) Reporting guidelines for terrestrial respirometry: building openness, transparency of metabolic and evaporative water loss data. Comp. Biochem. Physiol. Part A Mol. Integr. Physiol. 296: 111688. 10.1016/j.cbpa.2024.111688. 38944270

[ref344] Wu NC, Alton LA, Clemente CJ, Kearney MR, White CR (2015) Morphology and burrowing energetics of semi-fossorial skinks (*Liopholis* spp.). J Exp Biol 218: 2416–2426. 10.1242/jeb.113803. 26056244

[ref345] Wu NC, Bovo RP, Enriquez-Urzelai U, Clusella-Trullas S, Kearney M, Navas CA, Kong JD (2024a) Global exposure risk of frogs to increasing environmental dryness. Nat Clim Change 14: 1314–1322. 10.1038/s41558-024-02167-z.

[ref346] Wu NC, Cramp RL, Franklin CE (2017) Fixing a leaky skin: upregulation of ion transport proteins during sloughing. J Exp Biol 220: 2026–2035. 10.1242/jeb.151738. 28566357

[ref347] Wu NC, Cramp RL, Franklin CE (2018) Body size influences energetic and osmoregulatory costs in frogs infected with *Batrachochytrium dendrobatidis*. Sci Rep 8: 3739. 10.1038/s41598-018-22002-8. 29487313 PMC5829222

[ref348] Wu NC, Fuh N-T, Borzée A, Wu C-S, Kam Y-C, Chuang M-F (2025) Developmental plasticity to pond drying has carry-over costs on metamorph performance. Conserv. Physiol. 13: coaf008. 10.1093/conphys/coaf008. 39974208 PMC11839272

[ref349] Wu NC, McKercher C, Cramp RL, Franklin CE (2019) Mechanistic basis for the loss of water balance in green tree frogs infected with a fungal pathogen. Am J Physiol Regul Integr Comp Physiol 317: R301–R311. 10.1152/ajpregu.00355.2018. 31141416

[ref350] Wu Y, Miao C, Slater L, Fan X, Chai Y, Sorooshian S (2024) Hydrological projections under CMIP5 and CMIP6: sources and magnitudes of uncertainty. B Am Meterorol Soc 105: E59–E74. 10.1175/BAMS-D-23-0104.1.

[ref351] Yenmiş M, Ayaz D, Sherbrooke W, Veselý M (2024) Comparative analyses of micro-and macro-scale surface structures in the convergent evolution of rain-harvesting behaviour in lizards. J Zool 322: 58–75. 10.1111/jzo.13123.

[ref352] Zani PA, Stein SJ (2018) Field and laboratory responses to drought by common side-blotched lizards (*Uta stansburiana*). J Arid Environ 154: 15–23. 10.1016/j.jaridenv.2018.03.001.

[ref353] Zargar A, Sadiq R, Naser B, Khan FI (2011) A review of drought indices. Environ Rev 19: 333–349. 10.1139/a11-013.

[ref354] Zhang W, Zhou T, Wu P (2024) Anthropogenic amplification of precipitation variability over the past century. Science 385: 427–432. 10.1126/science.adp0212. 39052805

[ref355] Zhou X, Prigent C, Yamazaki D (2021) Toward improved comparisons between land-surface-water-area estimates from a global river model and satellite observations. Water Resour Res 57: e2020WR029256. 10.1029/2020WR029256.

[ref356] Zomer RJ, Xu J, Trabucco A (2022) Version 3 of the global aridity index and potential evapotranspiration database. Sci. Data 9: 409. 10.1038/s41597-022-01493-1. 35840601 PMC9287331

